# Efficacy and Safety of Glucagon‐Like Peptide‐1 Agonists for Psychiatric Symptoms: A Systematic Review

**DOI:** 10.1002/brb3.70661

**Published:** 2025-07-09

**Authors:** Shakila Meshkat, Corinna Di Luciano, Alyssa Swiderski, Gloria Li, Reinhard Janssen Aguilar, Benjamin T. Dunkley, Amy C. Reichelt, Yanbo Zhang, Andrew Greenshaw, Eric Vermetten, Rakesh Jetly, Satya Dash, Sri Mahavir Agarwal, Jennifer Swainson, Venkat Bhat

**Affiliations:** ^1^ Interventional Psychiatry Program St. Michael's Hospital Toronto Ontario Canada; ^2^ Neurosciences & Mental Health Hospital for Sick Children Research Institute Toronto Canada; ^3^ Institute of Medical Science University of Toronto Toronto Ontario Canada; ^4^ Department of Physiology and Pharmacology Western University London Canada; ^5^ School of Biomedicine, Adelaide Medical School University of Adelaide Adelaide South Australia Australia; ^6^ Department of Psychiatry University of Alberta Edmonton Alberta Canada; ^7^ Neuroscience and Mental Health Institute (NMHI) University of Alberta Edmonton Alberta Canada; ^8^ Department of Psychiatry Leiden University Medical Center Leiden the Netherlands; ^9^ The Institute of Mental Health Research University of Ottawa Royal Ottawa Hospital Ontario Canada; ^10^ Department of Endocrinology Toronto General Hospital and University of Toronto Toronto Ontario Canada; ^11^ Department of Psychiatry University of Toronto Toronto Ontario Canada; ^12^ Centre for Addiction and Mental Health Toronto Ontario Canada; ^13^ Neuroscience Research Program St. Michael's Hospital Toronto Ontario Canada

**Keywords:** depression, glucagon‐like peptide‐1 (GLP‐1) receptor agonists, mental disorders, psychiatric disorders, systematic review

## Abstract

**Background**: Glucagon‐like peptide‐1 receptor agonists (GLP‐1 RAs) have emerged as potential therapeutic options for psychiatric symptoms due to their effects on mood and behavior. However, their use has been primarily for metabolic diseases, and limited research has evaluated their psychiatric efficacy. This systematic review examined the impact of GLP‐1 RAs on psychiatric symptoms, categorizing studies based on whether psychiatric outcomes were primary or secondary.

**Methods**: A comprehensive search through OVID databases from inception to November 2024 identified 26 studies (*n* = 3020), with 5 studies evaluating psychiatric symptoms as primary outcomes (e.g., substance use disorders) and 21 studies assessing secondary outcomes across various conditions. Additionally, 10 registered clinical trials were identified.

**Results**: Among the five studies targeting primary outcomes, exenatide and dulaglutide were investigated in cocaine use disorder, alcohol use disorder, and nicotine dependence. Exenatide 2 mg once‐weekly demonstrated reductions in cocaine craving in two cases over 6 weeks in a case series study. However, mixed results were reported across studies, including no significant changes in a separate case series (*n* = 12, *p* = 0.46). Dulaglutide 1.5 mg weekly did not significantly affect smoking abstinence (RR = 0.87, *p* = 0.25) but reduced alcohol consumption by 29%   in a secondary analysis. For studies assessing secondary outcomes, GLP‐1 RAs showed variable effects. In mood disorders, liraglutide 1.8 mg/day significantly improved depression and anhedonia. In Type 2 diabetes, semaglutide improved diabetes‐related quality of life (Cohen' and anxiety (Cohen's *d* = 0.48) compared to dulaglutide. Exenatide studies reported mixed results, with one randomized controlled trial (RCT) showing significant reductions in Beck's Depression Inventory (BDI) scores (*Δ* = −5.2). A total of nine RCTs were included in this review and the risk of bias was assessed using the JBI Critical Appraisal Checklist for RCTs. All included RCTs met the majority of criteria, indicating moderate‐to‐high methodological quality. Of the included RCTs, three reported a statistically significant effect on the primary psychiatric outcome, whereas six reported no significant effect.

**Conclusion**: GLP‐1 RAs showed mixed and inconclusive effects on psychiatric symptoms. Although some studies suggested potential benefits, others reported null findings. Given the variability across trials, further research is needed to clarify their therapeutic potential.

## Graphical abstract

This systematic review synthesizes data from 26 studies (*n* = 3020) and 10 registered trials assessing the psychiatric effects of GLP‐1 receptor agonists (GLP‐1 RAs). Although evidence for substance use disorders was limited and mixed, GLP‐1 RAs showed modest‐to‐moderate benefits for mood symptoms, anxiety, and quality of life in comorbid conditions. Further targeted trials are warranted to clarify their psychiatric potential.

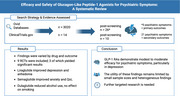

## 1 Introduction

Glucagon‐like peptide‐1 (GLP‐1) is a multifunctional hormone synthesized primarily in the L cells of the ileum, colon, and rectum, as well as in specific regions of the central nervous system (CNS) (Müller et al. 2019). Released in response to food intake, GLP‐1 activates GLP‐1 receptors (GLP‐1Rs), leading to decreased glucagon secretion, increased insulin secretion, appetite suppression, delayed gastric emptying, and regulation of glucose homeostasis (Meier and Nauck 2005). GLP‐1Rs are widely distributed in brain regions involved in cognition and emotion, including the prefrontal cortex, anterior cingulate cortex, hippocampus, amygdala, hypothalamus, and brainstem. Notably, they are present in the serotonin‐producing dorsal raphe nucleus and dopamine‐rich midbrain areas, highlighting their relevance to mood regulation and neuropsychiatric health (Gupta et al. 2023; Cabou and Burcelin 2011). GLP‐1R agonists (GLP‐1 RAs), originally developed for Type 2 diabetes mellitus (T2DM), have demonstrated CNS effects beyond glycemic control (Gupta 2013). These agents include short‐acting drugs like exenatide and lixisenatide and long‐acting drugs like liraglutide, dulaglutide, and semaglutide (Manandhar and Ahn 2015). Preclinical studies indicate that GLP‐1 RAs reduce neuroinflammation, enhance neurogenesis, improve synaptic plasticity, and modulate neurotransmitter systems implicated in psychiatric disorders (Erbil et al. 2019; Isacson et al. 2011). Given these effects, there is growing interest in their potential as therapeutic agents for conditions such as depression, anxiety, and cognitive impairment.

Psychiatric disorders are a significant global health burden, affecting millions and often leading to profound impacts on quality of life (Saarni et al. 2007). Traditional pharmacotherapies for depression and anxiety, including SSRIs, primarily target monoaminergic pathways, but are often limited by treatment resistance, delayed onset, and intolerable side effects (Penn and Tracy 2012). This has driven interest in alternative treatment pathways, including the gut‐brain axis and inflammatory signaling, which are implicated in the pathophysiology of psychiatric and neurological disorders (Mörkl et al. 2018). Emerging evidence suggests that GLP‐1 signaling may influence neuropsychiatric health through mechanisms related to inflammation, neurotransmitter balance, and metabolic regulation (Kim et al. 2020). Clinical studies have begun to explore the psychiatric effects of GLP‐1 RAs, with preliminary trials suggesting potential benefits in mood disorders and cognitive function (Acar and Erbaş1 2021; Robert et al. 2015; Richards et al. 2023; McIntyre et al. 2013). However, the extent to which these effects are primary (directly targeted) versus secondary (observed in metabolic trials) remains unclear.

This systematic review synthesizes the current evidence on the impact of GLP‐1 RAs on psychiatric symptoms, categorizing studies based on whether these symptoms were assessed as primary or secondary outcomes. This distinction is essential for evaluating the robustness and clinical relevance of reported effects. Primary outcome studies are designed specifically to investigate psychiatric effects, using validated assessment tools and adequate sample sizes. In contrast, secondary outcome studies often emerge from metabolic trials where psychiatric effects are incidental but provide insight into potential indirect mechanisms related to improved metabolic profile. By systematically analyzing these studies, this review aims to clarify the therapeutic potential of GLP‐1 RAs in psychiatry, identify research gaps, and provide direction for future investigations.

## 2 Materials and Methods

### 2.1 Search Strategy

This systematic review followed Preferred Reporting Items for Systematic reviews and Meta‐Analyses guidelines (Page et al. 2021). A comprehensive search was conducted in three databases (MEDLINE, PsycINFO, Embase) through OVID from inception until November 2024. No limitation on date or language was placed. We also searched the first 10 pages of Google Scholar and the references of relevant articles. The search for past and ongoing registered clinical trials was conducted on the ClinicalTrials.gov (https://www.clinicaltrials.gov/) platform on May 17, 2024. Search keywords included GLP‐1 RAs (semaglutide, exenatide, albiglutide, dulaglutide, liraglutide, lixisenatide, and loxenatide), psychiatric disorders (major depressive disorder (MDD), substance use disorders (SUDs), anxiety, binge eating disorder (BED), post‐traumatic stress disorder (PTSD), and bipolar disorder (BD), schizophrenia). The detailed search keywords are included in Table S1.

### 2.2 Screening Process and Inclusion Criteria

Studies were included if they meet the following inclusion criteria: (1) Original studies such as randomized controlled trial (RCT), case report, case series, preprints, cross‐sectional, case–control, cohort, uncontrolled observational, prospective and retrospective studies with or without a comparison group; (2) that used GLP‐1 RAs; (3) reported data on psychiatric disorders symptoms; and (4) in individuals with primary diagnosis of psychiatric disorders or other diseases such as diabetes or obesity. Studies investigating dementia, Parkinson's disease, and other neurological disorders were excluded as they were beyond the scope of this review. All included published studies had to be available in English, with translations of the full text permitted. Studies, including participants diagnosed with comorbid neurodevelopmental or neurocognitive disorders, such as attention‐deficit/hyperactivity disorder (ADHD), autism spectrum disorder (ASD), or Alzheimer's Disease, were excluded, as were those with somatic symptom disorders, sleep–wake disorders (including breathing‐related sleep disorders and parasomnias), sexual dysfunctions, gender dysphoria, and paraphilic disorders.

Furthermore, we excluded studies using datasets, conference abstracts, systematic reviews, animal studies, narrative reviews, meta‐analyses, and letters to the editor. Two authors (S.M. and C.D.) independently performed the title/abstract and full‐text screening on Covidence (https://www.covidence.org/) and screened articles for inclusion according to the eligibility criteria. Conflicts were resolved by discussion between the reviewers. Studies were categorized based on whether psychiatric symptoms were evaluated as primary or secondary outcomes to clarify their designation in studies investigating GLP‐1 RAs, in line with clinical outcome reporting conventions. This categorization allows for a clearer understanding of whether these symptoms were the main focus of intervention or observed as secondary benefits, aiding in the interpretation of the therapeutic potential for psychiatric disorders.

### 2.3 Data Extraction

Two reviewers (S.M. and C.D.) independently reviewed the full text of the included articles and extracted the following data: author, publication year, country, study design, intervention, diagnosis, outcome, outcome measure, number of participants, and results. All outcome measures were recorded along with the protocol for each. We also extracted the following variables from registered clinical trials: title, study URL, study status, registration year, primary diagnosis, country, type of funding, mean age, number of participants, % of female participants, actual enrollment, number of participants that completed treatment, number of participants that completed the entire trial, inclusion of healthy participants, allocation, intervention model, masking, study, number of treatment arms, description of study arms, intervention description, study period, drug used, outcomes and outcome measures. For the studies that had results posted, results and adverse effects (AEs) were extracted. The descriptive statistical analysis was performed using Excel and GraphPad Prism, and data visualization was performed using Excel and GraphPad Prism.

### 2.4 Quality Assessment

All published studies included in this review were assessed for quality using the JBI Critical Appraisal Tools Checklist for systematic reviews by two independent assessors (A.S. and C.D.). The JBI checklists used included those for RCTs, case series, case reports, and cohort studies (Porritt et al. 2014).

## 3 Results

### 3.1 Search Results

**FIGURE 1:**
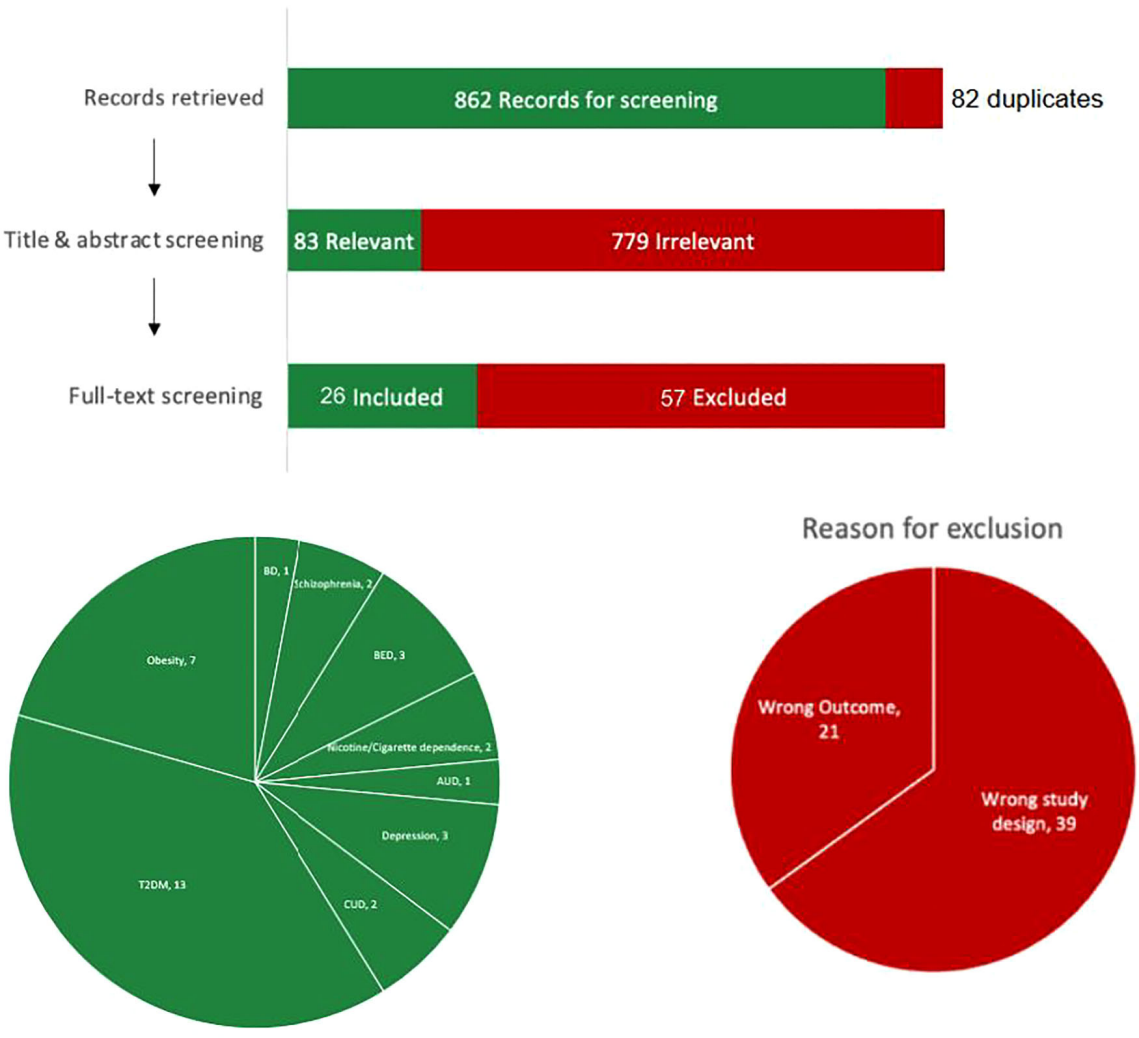
PRISMA flow chart.

**FIGURE 2:**
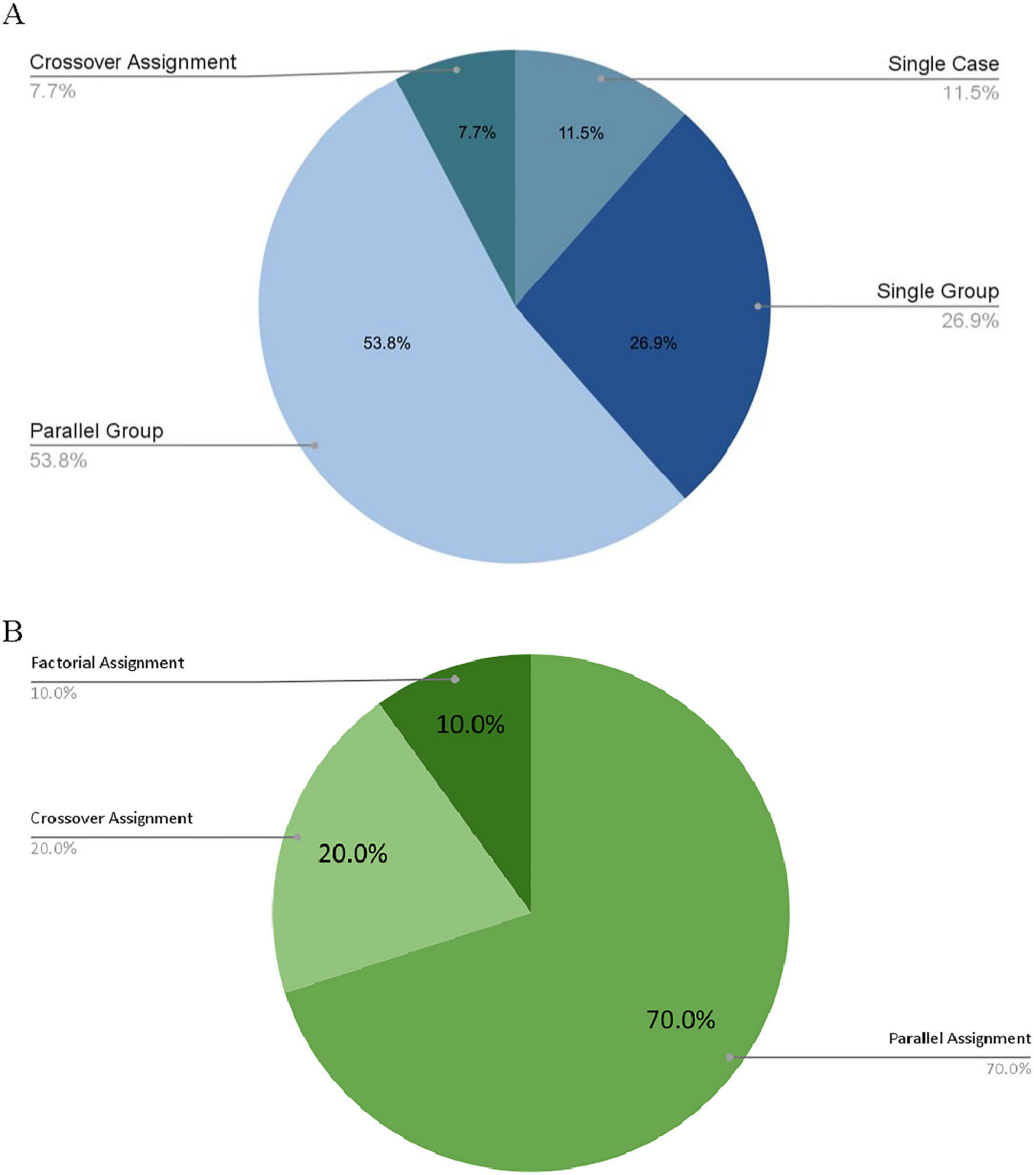
(A) Published papers by intervention model. (B) Intervention model used in included unpublished clinical trials.

**FIGURE 3:**
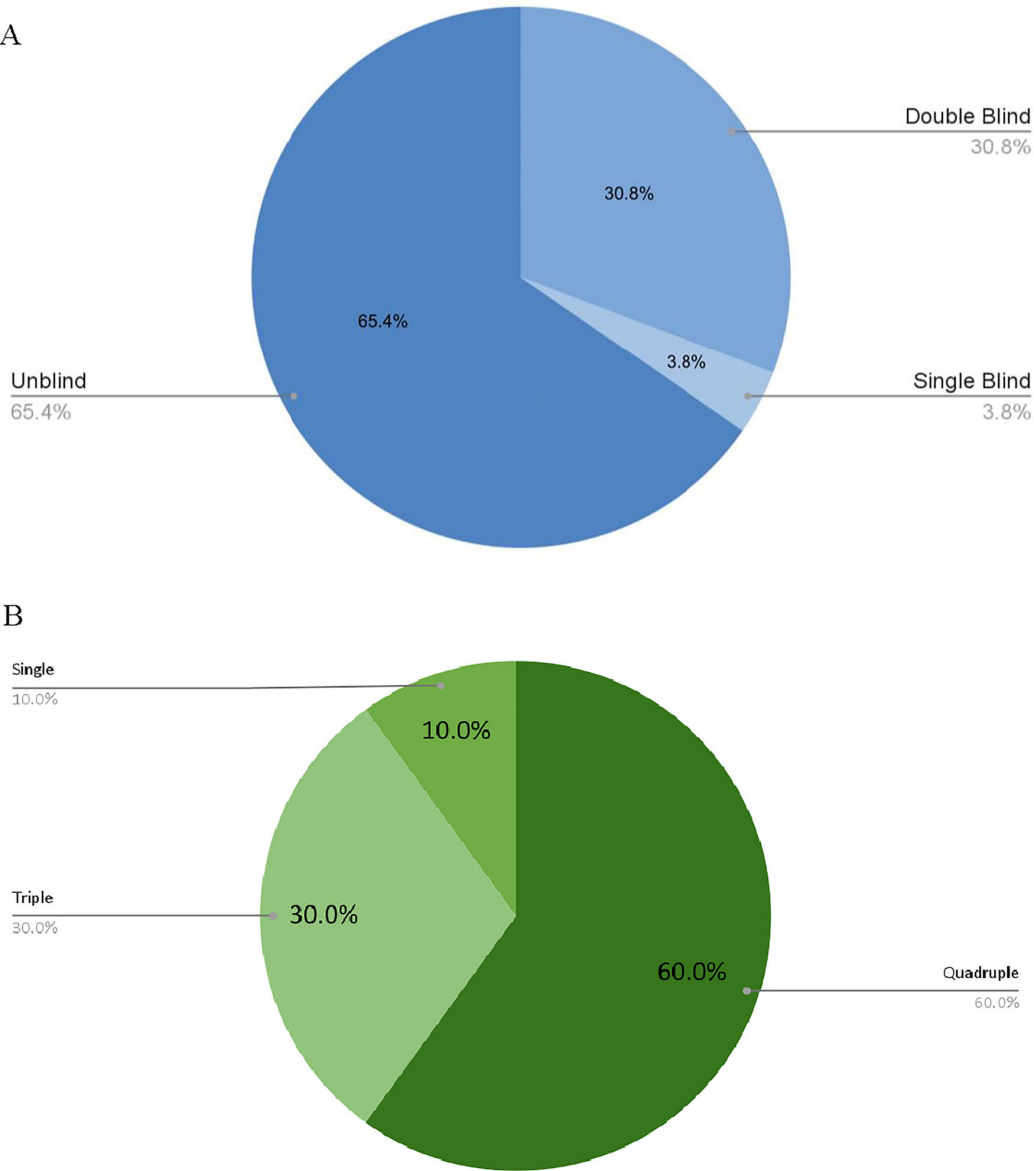
(A) Masking employed in published papers. (B) Masking employed in unpublished clinical trials.

**FIGURE 4:**
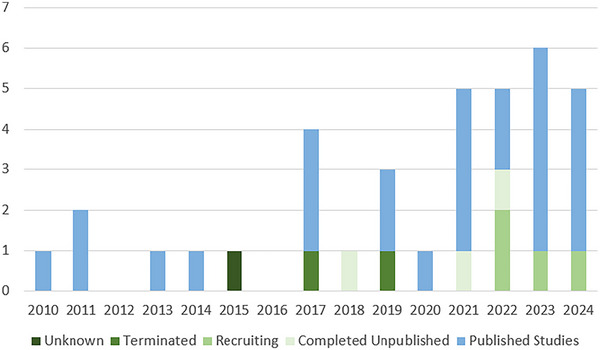
Published studies and registered trials by publication year.

**FIGURE 5:**
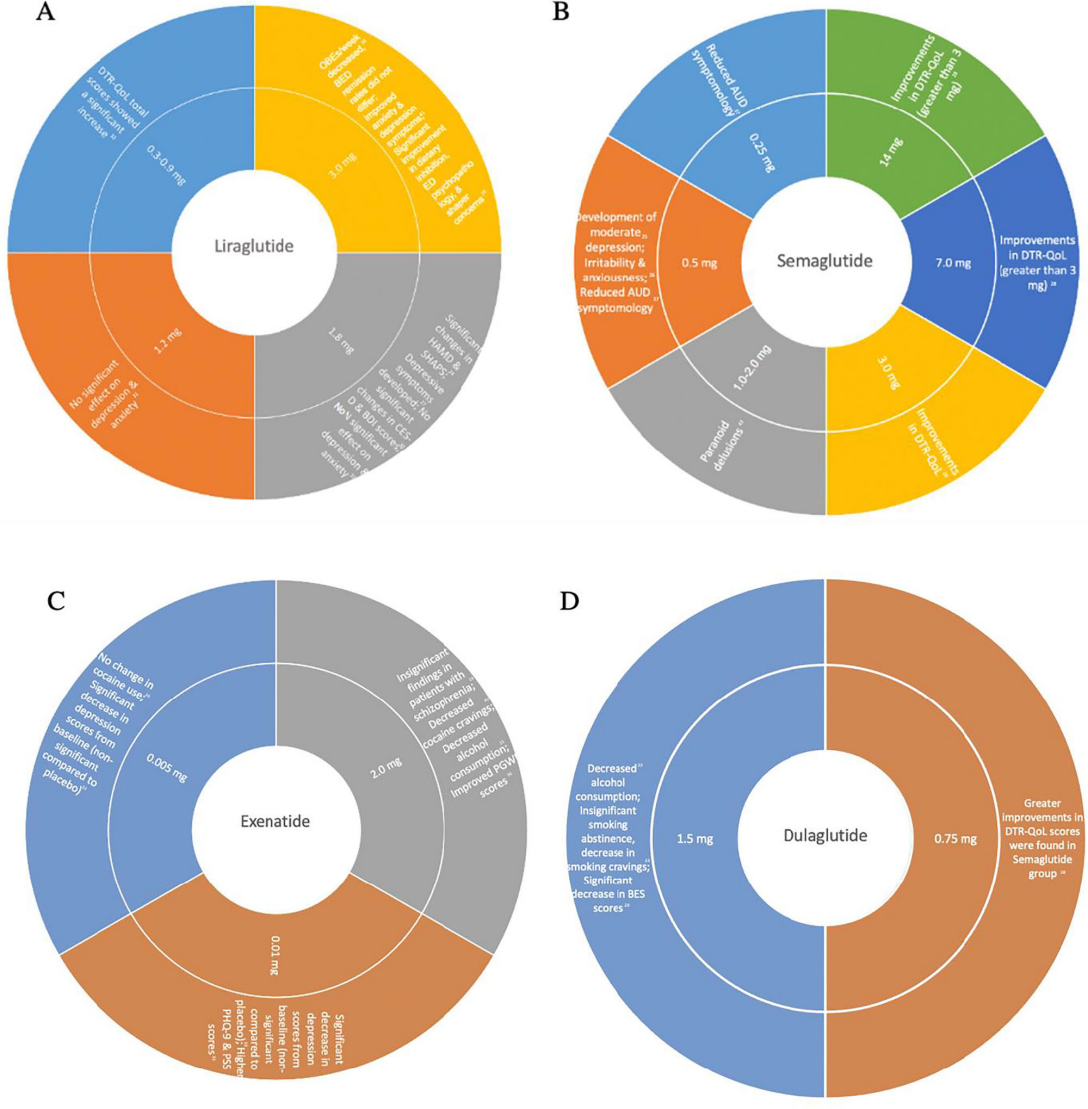
GLP‐1 RAs effects on psychiatric symptoms.(A) Studies involving Liraglutide. (B) Studies involving Semaglutide. (C) Studies involving Exenatide. (D) Studies involving Dulaglutide. AUD, alcohol use disorder; BDI, Beck's Depression Inventory; BED, binge eating disorder; BES, binge eating score; CES‐D, Center for Epidemiologic Studies Depression Scale; DTR‐Qol, diabetes therapy‐related quality‐of‐life; PGWB, psychological general well‐being; PHQ‐9, Patient Health Questionnaire‐9; PSS, Perceived Stress Scale.

**FIGURE 6:**
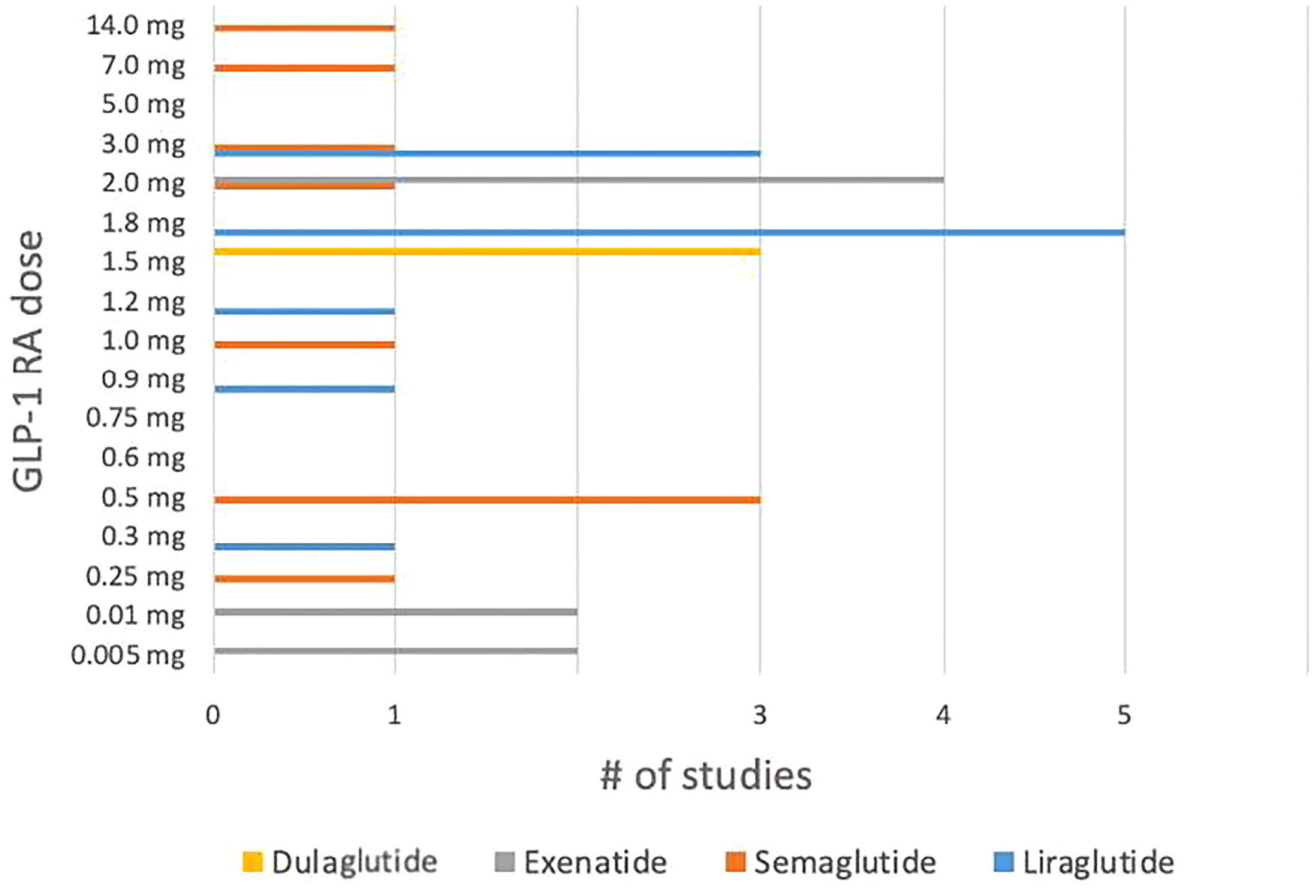
Doses of GLP‐1 RAs administered across published studies. GLP‐1 RA, Glucagon‐like peptide‐1 receptor agonist.

The search of the OVID databases yielded 945 records. After the removal of duplicates (*n* = 82), 862 records were included in the first‐level screening. Following the screening of the titles and abstracts, 779 studies were excluded. We reviewed the full text of the remaining papers (*n* = 83). Out of the 83 papers, a total of 26 studies involving 3020 participants were included in this systematic review (Yammine et al. 2023; Angarita et al. 2021; Klausen et al. 2022; Lüthi et al. 2024; Probst et al. 2023; Mansur et al. 2017; Li et al. 2023; Rajaram and Madan 2024; He et al. 2024; Ishii et al. 2021; Da Porto et al. 2020; De Wit et al. 2016; Bode et al. 2010; Ishii et al. 2017; Idris et al. 2013; Grant et al. 2011; Eren‐Yazicioglu et al. 2021; Best et al. 2011; Richards et al. 2023; Allison et al. 2023; Chao et al. 2019; Kahal et al. 2019; Apperley et al. 2021; Ishøy et al. 2017; Hejdak et al. 2024; Drew et al. 2022; Clément et al. 2004). The remaining 57 papers were excluded due to wrong study design (*n* = 36) and wrong outcome (*n* = 21) (Figure 1). Additionally, 10 registered RCTs were identified through clinicaltrials.gov, 3 of which provided study results. The full characteristics of the included studies and clinical trials are indicated in Tables 1 and 2, Figures 2, 3, 4, 5, and 6. Quality assessment results can be found in Table S2.

**TABLE 1:** Treatment parameters and outcome measures of published studies.

Author, year	Country	Study design	No. of participants	Intervention	Diagnosis	Outcome	Outcome measures	Results
**Studies with primary outcome of psychiatric symptoms**								
Yammine et al. (2023)	USA	Case series	3	Exenatide 2 mg once‐weekly for 6 weeks	CUD	Cocaine use^a^, mood	The brief substance craving scale, BDI‐II, PANAS, craving intensity	Case A rated craving as moderate to extremely intense across weeks 1 to 5, before declining to slightly at Week 6. Case B reported moderate craving intensity at baseline, which decreased to none across Weeks 3 to 6. Case C reported slight craving intensity at baseline, which fluctuated between moderate and none across Weeks 1 to 6
Angarita et al. (2022)	USA	RCT	13	Exenatide 5 µg, 0.02 mL single dose	CUD	Cocaine use	Number of infusions	Pre‐treatment with exenatide (8.5 ± 1.2) did not change the number of infusions in comparison to pre‐treatment with the placebo (9.1 ± 1.2) (*F*(1, 12) = 0.76, *p* = 0.39)
Klausen et al. (2022)	Denmark	RCT	127	Exenatide 2 mg once‐weekly for 26 weeks	AUD	Alcohol use	Reduction in heavy drinking days, DUDIT, AUDIT	For both groups, the number of heavy drinking days and total alcohol intake were strongly reduced, but there were no significant differences between the two groups. The exenatide group had a worsening in DUDIT score of 0.96 points (95% CI, 0.7–1.3, *p *< 0.001) relative to the placebo group
Luthi et al. (2024)	Switzerland	RCT	225	Dulaglutide 1.5 mg once‐weekly mg for 12 weeks	Nicotine dependence	Long‐term smoking abstinence/point prevalence abstinence rate	Self‐reported, abstinence rates, prolonged abstinence rates, and smoking reduction	Of the study participants, 63% (*n* = 80/127) (dulaglutide group) and 65% (*n* = 83/128) (placebo group) were abstinent after 12 weeks. These abstinence rates declined to 43% (*n* = 54/127) and 41% (*n* = 52/128), respectively, after 24 weeks and to 32% (*n* = 41/127) and 32% (*n* = 41/128), respectively, after 52 weeks
Probst et al. (2023)	Switzerland	RCT	151	Dulaglutide 1.5 mg once‐weekly mg for 12 weeks	Nicotine dependence	Total consumption of alcohol per week	Number of glasses of each type of alcohol consumed per week	At Week 12, participants receiving dulaglutide drank 29% less (relative effect = 0.71, 95% CI 0.52–0.97, *p* = 0.04) than participants receiving placebo
**Studies with secondary outcome of psychiatric symptoms**								
Mansur et al. (2017)	Canada	Open‐label trial	17	Liraglutide 1.8 mg daily for 4 weeks	MDD or BD	Depression, anhedonia	HAMD SHAPS	Significant changes in HAMD (mean = 12.18, SD 4.82 vs. 8.41, SD 6.12, Cohen's *d* 0.68, *p* = 0.022) and SHAPS (mean = 38.35, SD 6.41 vs. 42.88, SD 7.73, *p* = 0.010)
Li et al. (2023)	China	Case report	2	Semaglutide 0.5 mg weekly	Depression, T2DM	Depression severity	Mental state examination	Case 1 developed moderate depression after semaglutide, which improved after 1 week of cessation; Case 2 developed severe depression after 1 month of taking semaglutide, and after 1 week of cessation, the patient's mood improved
Manoharan et al. (2024)	USA	Case Report	1	Semaglutide 0.5 mg	Depression, T2DM	Mood change following semaglutide initiation	Self‐reported changes in mood	After 4 weeks of treatment with semaglutide, the patient reported feeling more irritable and anxious than usual. Semaglutide was then discontinued, and over the following several weeks, her mood and symptoms improved
Hejdak et al. (2024)	USA	Case report	1	Semaglutide 1–2 mg/weekly	Schizophrenia, T2DM	Psychological/schizophrenia symptoms	Schizophrenia symptoms	Two to 3 weeks after the initiation of semaglutide, the patient began experiencing paranoid delusions. The delusions were reduced due to an increase in Geodon dosage. The patient maintained stability until the semaglutide dosage increased 7 months later. Delusions resolved approximately 1–2 weeks thereafter. Approximately 2 weeks later, the patient had the same return of paranoid delusions
Richards et al. (2023)	USA	Retrospective cohort study	48	Semaglutide	Obesity, BED	Binge eating	BES scores	Patients receiving semaglutide only exhibited greater reductions in BES scores compared to the other groups. Combined pharmacotherapy with both semaglutide and the other anti‐obesity medications did not result in greater reductions in BES scores compared to the semaglutide‐only group
Allison et al. (2021)	USA	RCT	27	Liraglutide 3.0 mg/day for 12 weeks	Obesity, BED	Binge eating, depression	OBEs/week, EDE, PHQ‐9	At Week 17, OBEs/week decreased by 4.0, 0.6 in liraglutide participants and 2.5, 0.5 in placebo participants (*p* = 0.37, mean difference = 1.2). BED remission rates of 44% and 36%, respectively, did not differ. Changes in eating behavior, depressive symptoms, and quality of life did not differ between the groups
Ishoy et al. (2017)	Denmark	RCT	45	Exenatide 2 mg weekly for 3 months	Schizophrenia, obesity	Neurocognition, schizophrenia symptom severity	BACS, SF‐36, PSP, PANSS	Repeated measures analysis of variance on BACS composite score showed a significant effect of “time” (*p *< 0.001), no effect of “group” (*p* = 0.64) and no “time × group” interaction (*p* = 0.77). For SF‐36, PSP and PANSS, only significant “time” effects were found
Da Porto et al. (2020)	Italy	Pilot trial	60	Dulaglutide 1.5 mg weekly for 12 weeks	T2DM, BED	Binge eating	Italian version of the BES questionnaire	BES score (12,067 vs. 0467 *p *< 0.0001) were significantly decreased only by treatment with dulaglutide. Multivariate regression analysis showed that decrease in the BES score was independently and directly related to changes in body weight (*p *< 0.0001) and HbA1c (*p* ¼ 0.033) obtained independently of the type of treatment
De Wit et al. (2014)	Netherlands	Open label trial	18	Liraglutide 1.8 mg/day for 26 weeks	T2DM	Depression	BDI	No significant changes in BDI score (−1 ± 2 vs. 0 ± 1, *p* = 0.46) neither among nor between the two groups during treatment
Idris et al. (2013)	United Kingdom	Non randomized clinical trial	16	Exenatide 5–10 µg twice daily for 22 weeks	T2DM	Depression	BDI	There was a significant reduction in depression scores from baseline to exenatide groups (*p* = 0.001), but a non‐significant reduction in depression scores between placebo and exenatide
Ishii et al. (2021)	Japan	RCT	458	Semaglutide 3, 7, or 14 mg/day, or weekly dulaglutide 0.75 mg	T2DM	Changes in health‐related quality of life including anxiety	DTR‐QoL	Semaglutide, particularly at 7 and 14 mg doses, showed greater improvements in DTR‐QoL total scores compared to dulaglutide at Week 52. Semaglutide was also associated with better sustained improvements over time, with less decline in DTR‐QoL scores from Weeks 26 to 52 compared to dulaglutide
Ishii et al. (2017)	Japan	Observational	204	Liraglutide 0.3–0.9 mg/day for 12 weeks	T2DM	Changes in health‐related quality of life including anxiety	DTR‐QoL	At Week 12, the DTR‐QoL total scores showed a significant increase from 61.9 ± 16.2 at baseline to 69.7 ± 16.8 (*p *< 0.001). Significant improvement was also apparent throughout the four domains, reflecting improvements in anxiety, treatment satisfaction, and daily activity burden
Bode et al. (2010)	USA	RCT	732	Liraglutide 1.2 or 1.8 mg daily for 52 weeks	T2DM	Depressive and anxiety symptoms	HRQoL	No significant effect of liraglutide on depression and anxiety
He et al. (2024)	China	Case report	1	Liraglutide 1.8 mg qd	T2DM	Depressive symptoms	HAMD	Depressive symptoms significantly improved within a week after discontinuing liraglutide and starting antidepressant therapy. At discharge, the patient's HAMD was 6, and Hamilton Anxiety Scale score was 5. A 2‐week follow‐up showed that the patient's mood remained stable
Eren‐Yazicioglu et al. (2021)	Turkey	Cross‐sectional	43	Exenatide 10 µg twice daily for at least 3 months	T2DM	Depressive and anxiety symptoms	PHQ‐9, PSS	Patients on exenatide had higher PHQ‐9 (9.70 ± 4.92 vs. 6.70 ± 4.66; *p* = 0.026), and PSS (29.39 ± 6.70 vs. 23.35 ± 7.69; *p* = 0.015) scores. Those on exenatide reported higher PSS, with higher PSS being associated with higher PHQ‐9 levels (*b* = 0.236)
Best et al. (2011)	USA	Double‐dummy	491	Exenatide 2 mg weekly for 26 weeks	T2DM	Psychological well‐being including anxiety and depression	PGWB	At Week 26, all three groups showed improvements in PGWB scores. No evidence found statistically significant differences between the exenatide and the other groups
Grant et al. (2011)	United Kingdom	Cohort	138	Exenatide for 6 months	T2DM	Anxiety and depression	HADS	At 6 months, treatment satisfaction (*p* < 0.05) and well‐being (*p* < 0.05) scores were greater, and the HADS scores (*p* < 0.05) significantly reduced in the exenatide‐treated patient group when compared with the insulin‐treated group
Richards et al. (2023)	USA	Case series	6	Semaglutide 0.25 or 0.5 weekly	Obesity	Alcohol use	AUDIT	All six identified patients (100%) had a significant reduction in AUD symptomatology based on AUDIT score improvement following treatment with semaglutide (mean decrease of 9.5 points, *p *< 0.001)
Kahal et al. (2019)	UK	Cross‐sectional	36	Liraglutide 1.8 mg weekly for 6 months	Obesity and PCOS	Depression	CES‐D	There was no significant change at 6 months in the number of women who scored 16 on the CES‐D questionnaire, suggestive of depression, in the PCOS group (baseline vs. 6‐month): 6 (32%) vs. 5 (26%), *p* ¼ 0.72; and controls: 5 (29%) vs. 3 (18%), *p* ¼ 0.42, respectively
Apperley et al. (2021)	UK	Clinical trial	7	Liraglutide 3.0 mg for 3 months	Obesity	Anxiety, depression	Child anxiety and depression scale	Anxiety and depressive symptoms showed improvement over the 3‐month intervention period. This effect was particularly prevalent in features of separation anxiety disorder. The mean difference over the time‐period for total anxiety and depression was not quite significant (95% CI from −1.11 to 22.11; *p* = 0.068)
Chao et al. (2019)	USA	RCT	150	Liraglutide 3.0 mg for 12 weeks	Obesity	Eating disorder psychopathology	EDE‐Q	At 24 weeks, groups receiving liraglutide (IBT‐liraglutide and multicomponent) showed significant improvements in dietary disinhibition, eating disorder psychopathology, and shape concerns compared to IBT‐alone. The multicomponent group reported greater reductions in binge eating episodes compared to the IBT‐alone group

Abbreviations: AUD, alcohol use disorder; AUDIT, Alcohol Use Disorders Identification Test; BACS, Brief Assessment of Cognition in Schizophrenia; BD, bipolar disorder; BDI, Beck's Depression Inventory; BED, binge eating disorder; BES, binge eating scale; CES‐D, Center for Epidemiologic Studies Depression Scale; CUD, cocaine use disorder; DTR‐QoL, diabetes therapy‐related quality‐of‐life; DUDIT, Drug Use Disorders Identification Test; EDE, eating disorder examination; EDE‐Q, eating disorder examination questionnaire; HADS, Hospital Anxiety and Depression Scale; HAMD, Hamilton Depression Rating Scale; HRQoL, Health‐related quality of life; MDD, major depressive disorder; OBEs, objective binge episodes; PANAS, Positive and Negative Affect Schedule; PANSS, Positive and Negative Syndrome Scale; PCOS, polycystic ovary syndrome; PGWB, psychological general well‐being index; PHQ‐9, Patient Health Questionnaire‐9; PSP, Personal and Social Performance Scale; PSS, Perceived Stress Scale; RCT, randomized controlled trial; SF‐36, Short Form Health Survey; SHAPS, Snaith–Hamilton Pleasure Scale; T2DM, Type 2 diabetes mellitus.

^a^ Indicates the primary outcome for studies that reported on multiple outcomes.

**TABLE 2:** Treatment parameters and outcome measures of clinical trials.

NCT number	Study status	# of participants	Allocation	Intervention model	Masking	Drug	Outcome	Clinical indication	Outcome measures
NCT03279731	Terminated	36	Randomized	Parallel assignment	Quadruple	Liraglutide	Binge eating episodes^a^, symptoms, and remission	BED	BE, RBE, IBS, CBW
NCT05895643	Recruiting	108	Randomized	Parallel assignment	Quadruple	Semaglutide	Change in heavy drinking days/alcohol consumption^a^, days without alcohol, and relapse	AUD	HDD; TAC, DWA, TR, RLAC, PACS, AUDIT, DUDIT, FIB4, WHOQOL‐BREF, FTND, GGT, ALAT, PEth, MCV, BW, BW, GP; GABA levels; fMRI‐ACR
NCT03645408	Terminated	3	Randomized	Crossover assignment	Quadruple	Exenatide	Alcohol consumption^a^ and alcohol cravings	AUD	AC
NCT02690987	Unknown	95	Randomized	Crossover assignment	Quadruple	Exenatide	Brain activation during cigarette, alcohol, and food picture evaluation task^a^, brain activation during negative emotional reactivity task, salience resting state, limbic resting state, default mode resting state, alcohol and nicotine craving	NUD, AUD	fMRI‐WMR, VAS), AUQ, QSU, VAS, progressive ratio task breakpoint, plasma glucose, insulin, cortisol, growth hormone, GLP1, peptide YY, exenatide and desacyl ghrelin concentrations, CGM, Smoking relapse rate, alcohol relapse rate
NCT04199728	Completed	27	Randomized	Parallel assignment	Quadruple	Liraglutide	Change in self‐reported cue‐elicited drug craving^a^, change in ambient drug craving^a^, change in blood pressure, change in heart rate, change in respiratory rate, change in body weight, and percent change in body weight	OUD	Body weight; percent change in body weight; blood oxygenation level response to visual opioid drug cues in prefrontal cortex using fNIRs; blood oxygenation level response to visual opioid drug cues in prefrontal cortex using fNIRs measures regional cerebral oxygenation saturation, VAS, heart rate, respiratory rate; HbA1c; fructosamine levels
NCT05892432	Recruiting	135	Randomized	Factorial assignment	Quadruple	Semaglutide	Alcohol cue‐elicited craving^a^ and alcohol consumption	AUD	Change in cue craving visual analog score; number of drinks per day; percentage of heavy drinking days
NCT05520775	Completed	48	Randomized	Parallel assignment	Triple blind	Semaglutide	Alcohol consumption^a^, change in subjective stimulation from alcohol, change in subjective sedation from alcohol, alcohol demand, cigarette demand, daily alcohol use, daily cigarette use	AUD	Volume of alcohol consumed; breath alcohol concentration; subjective stimulation from alcohol, subjective sedation from alcohol, alcohol demand, cigarette demand, daily alcohol use, daily cigarette use, weight; HbA1c; alcohol elimination
NCT03712098	Completed	40	Randomized	Parallel assignment	Triple blind	Liraglutide	Nicotine usage/smoking abstinence^a^ and change in body weight	NUD	Number of participants with 7‐day point prevalence smoking abstinence at 12 weeks post‐target quit date; number of participants with 7‐day point prevalence smoking abstinence at 26 weeks post‐target quit date; body weight at 12 weeks post‐target quit date; body weight at 26 weeks post‐target quit date; calories consumed per day
NCT05333003	Recruiting	92	Randomized	Parallel assignment	Single blind	Semaglutide	Weight change^a^, psychopathology, quality of life, cognition, and nicotine dependence	Schizophrenia	Weight change; BMI; waist circumference; oral glucose tolerance test; visceral and hepatic adiposity; fasting lipid profile; BPRS; psychopathology—CDSS; GAF; CGI; change in cognitive performance; AQoL; WHODAS 2.0; IPAQ; the FTND; Penn state nicotine dependence index‐cigarette/electronic cigarette; C‐DHQ II;—FCQ; structural MRI; rsfMRI; ASL; 1H‐MRS
NCT05530577	Recruiting	48	Randomized	Parallel assignment	Triple blind	Semaglutide	Nicotine usage and reinstatement duration^a^, change in daily cigarette smoking, nicotine craving, subjective response to cigarette smoking, and change in body weight	TUD	Change in nicotine self‐administration; change in nicotine reinstatement duration; change in daily cigarette smoking; change in cigarette craving; change in subjective responses to cigarette smoking; change in body weight; change in HbA1c

Abbreviations: AQoL, assessment of quality of life; ASL, arterial spin labeling; AUD, alcohol use disorder; AUDIT, alcohol use disorders identification test; AUQ, alcohol urge questionnaire; BED, binge eating disorder; BMI, body mass index; BPRS, Brief Psychiatric Rating Scale; C‐DHQ II, Canadian Diet History Questionnaire II; CDSS, Calgary Depression Scale for Schizophrenia; CGI, clinical global impression scale; CGM, Cambridge gambling task risk taking measure; DUDIT, drug use disorders identification test; FCQ, food cravings questionnaire; fNIRs, functional near infrared spectroscopy; FTND, Fagerstrom Test of Nicotine Dependence; GAF, global assessment of functioning; IPAQ, International Physical Activity Questionnaire; MRS, magnetic resonance spectroscopy; NUD, nicotine use disorder; OUD, opioid use disorder; QSU, questionnaire of smoking urges; rsfMRI, resting state functional MRI; TUD, tobacco use disorder; VAS, visual analog scale; WHODAS 2.0, WHO Disability Assessment Schedule 2.0.

^a^ Indicates the primary outcome for studies that reported on multiple outcomes.

### 3.2 Published Studies With Primary Outcome of Psychiatric Symptoms

A total of five studies have investigated the effects of exenatide and dulaglutide in SUDs. Among these, three were RCTs (60%) and two were case series (40%). Sample sizes ranged from 3 participants in the smallest case series to 225 participants in the largest RCT. Exenatide was used in four studies (80%), whereas dulaglutide was used in two studies (40%). The conditions studied included cocaine use disorder (CUD) in three studies (60%), alcohol use disorder (AUD) in one study (20%), and nicotine dependence in two studies (40%). The studies utilized various GLP‐1 RAs at different doses and treatment durations. Liraglutide was administered at a dose of 1.8 mg daily for a duration of 4 weeks. Exenatide was used in multiple regimens: 2 mg once‐weekly for 6 weeks, 2 mg once‐weekly for 26 weeks, and a single dose of 5 µg (0.02 mL). Dulaglutide was administered at 1.5 mg once‐weekly, with an average treatment duration of 12 weeks.

#### 3.2.1 Substance Use Disorders

##### 3.2.1.1 Cocaine Use Disorder

In one RCT, Angarita et al. (2021) investigated a single dose of exenatide 5 µg in individuals with CUD, showing no significant changes in primary subjective outcomes for cocaine‐induced euphoria (“high,” 4.4 ± 0.8 vs. 4.0 ± 0.8; *F*(1, 12) = 1.73, *p* = 0.21) or craving (5.5 ± 0.9 vs. 5.4 ± 0.9; *F*(1, 12) = 0.58, *p* = 0.46) compared to placebo. A subsequent case series by Yammine et al. (2023) evaluated the 6‐week efficacy of exenatide 2 mg once‐weekly on cocaine use and mood in three patients with CUD. Cases A and C had cocaine‐positive urine drug screens (UDS) each week, with Case A using cocaine on 50% of days per week and Case C on more than 50%. In contrast, Case B tested positive in Weeks 1–3 but negative in Weeks 4–6. By Week 6, Case A's craving severity decreased from moderate/extreme to slight, Case B's diminished to none by Week 3, and Case C's fluctuated. Case A's Beck's Depression Inventory (BDI)‐II score decreased from moderate to minimal by Week 6, whereas Cases B and C's scores remained below clinical levels. Positive and negative affect scores were stable or improved across cases.

Although RCT data did not demonstrate significant changes in drug‐related subjective outcomes, the small case series data suggested potential reductions in cravings and depressive symptoms.

##### 3.2.1.2 Alcohol Use Disorder

Klausen et al. (2022) evaluated efficacy of exenatide 2 mg for AUD in an RCT of 127 patients. The results indicated no significant difference in the number of heavy drinking days between exenatide (*n* = 62) and placebo (*n* = 65) groups. The exenatide group had a worsening in Drug Use Disorders Identification Test (DUDIT) score of 0.96 points (95% CI, 0.7 to 1.3, *p* *<* 0.001) relative to the placebo group. At 6‐month follow‐up, the exenatide group had a higher AUDs Identification Test (AUDIT) score (5.1 points; 95% CI, 0.9 to 9.3, *p* = 0.02) than the placebo group (Klausen et al. 2022). Overall, exenatide did not reduce alcohol consumption and was associated with worsening substance use scores.

##### 3.2.1.3 Nicotine dependence

Luthi et al. (2024) evaluated the effect of 12‐week treatment with dulaglutide 1.5 mg once‐weekly, in addition to standard smoking cessation therapy (varenicline 2 mg/day plus behavioral counseling), on smoking abstinence rates in individuals with nicotine dependence. Smoking reduction, defined as a CO reduction of more than 50% or fewer cigarettes per day, was similar between the dulaglutide and placebo groups at Weeks 24 and 52. Point prevalence abstinence rates declined from nearly two‐thirds at week 12%–43% (54/127) in the dulaglutide group and 41% (52/128) in the placebo group at Week 24, reaching 32% in both groups by Week 52. Prolonged abstinence rates at Week 24 were 39% (50/127) in the dulaglutide group and 40% (51/128) in the placebo group, with rates of 29% (37/127) and 27% (35/128) by Week 52 (Lüthi et al. 2024). Overall, there was no significant difference in abstinence rates between the dulaglutide and placebo groups (63% vs. 65% at Week 12), and craving for smoking decreased similarly in both groups (Lüthi et al. 2024). In a secondary analysis, Probst et al. (2023) used data from the Luthi et al. study to evaluate changes in alcohol consumption after 12 weeks of dulaglutide treatment compared to placebo. The mean reduction in weekly alcohol consumption was −1.4 (SD 3.7) glasses in the dulaglutide group and −0.1 (SD 6.3) in the placebo group. At Week 12, participants in the dulaglutide group consumed an estimated 29% less alcohol (adjusted relative effect = 0.71, 95% CI 0.52–0.97, *p* = 0.04) than those in the placebo group. No interaction was found between baseline alcohol consumption and treatment (*p* = 0.2), indicating that the dulaglutide effect on alcohol consumption was independent of baseline intake. Adjusting for education level further increased the effect size, with dulaglutide participants drinking 36% less than placebo participants by Week 12 (relative effect = 0.64, 95% CI 0.47–0.86, *p* = 0.004) (Probst et al. 2023). Furthermore, abstinence rates from cigarettes at Week 12 among alcohol consumers were higher compared with nondrinkers (*n* = 114/159 [72%] vs. 51/96 [53%]; difference in proportions: −0.19 [95% CI −0.32 to −0.06]; *p* = 0.004) (Probst et al. 2023).

Overall, dulaglutide did not significantly enhance smoking cessation outcomes but showed a modest effect on reducing alcohol consumption in secondary analyses.

### 3.3 Published Studies With Secondary Outcome of Psychiatric Symptoms

A total of 21 studies have examined the effects of GLP‐1 RAs across various metabolic and psychiatric conditions, including T2DM, obesity, mood disorders, and schizophrenia. Among these, 29% (six studies) were RCTs, whereas the remaining included open‐label trials (14%, three studies), cross‐sectional studies (10%, two studies), cohort studies (10%, two studies), case reports (19%, four studies), and other observational designs (19%, four studies). Sample sizes varied widely, from single participants in case reports to up to 732 participants in the largest RCT. Liraglutide was the most frequently used GLP‐1 RA, featured in 38% (eight studies) of studies, followed by semaglutide in 29% (six studies), exenatide in 19% (four studies), and dulaglutide in 14% (three studies). Psychiatric symptoms studied included binge eating (29%, six studies), depression (24%, five studies), schizophrenia symptoms (10%, two studies), and anxiety symptoms (10%, two studies). The dosing and treatment durations across the 21 studies varied significantly. For liraglutide, doses ranged from 0.3 to 3.0 mg/day, with an average dose of approximately 1.8 mg/day. Treatment durations for liraglutide spanned from 4 weeks to 52 weeks, with an average duration of around 20 weeks. Semaglutide was administered at doses ranging from 0.25 mg weekly to 14 mg daily, with the most common doses being 0.5 mg weekly and 1–2 mg weekly. The treatment durations for semaglutide typically ranged from 12 weeks to 6 months. Exenatide was used at doses of 5–10 µg twice daily to 2 mg weekly, with treatment durations from 3 to 6 months, averaging approximately 20 weeks. Dulaglutide was most commonly used at a dose of 1.5 mg weekly for 12 weeks. Overall, the most common dosing regimens involved either daily or weekly administration, and the average treatment duration across all studies was around 16–20 weeks.

#### 3.3.1 Mood Disorders

Mansur et al. (2017) conducted an open‐label trial in 19 individuals with MDD or BD to evaluate the effect of liraglutide 1.8 mg/day on depression and anhedonia. Their results indicated significant changes in Hamilton Depression Rating Scale (HAMD) (mean = 12.18, SD 4.82 vs. 8.41, SD 6.12, Cohen's *d* 0.68, *p* = 0.022) and Snaith–Hamilton Pleasure Scale (SHAPS) (mean = 38.35, SD 6.41 vs. 42.88, SD 7.73, *p* = 0.010) (Mansur et al. 2017). Preliminary data suggested potential antidepressant effects of liraglutide, though findings were limited by small sample sizes and lack of control groups.

#### 3.3.2 Type 2 Diabetes

The psychiatric effects of GLP‐1 RAs in T2DM patients have shown significant variability. RCTs such as those by Ishii et al. (2021) and Da Porto et al. (2020) have explored the impact of GLP‐1 RAs on quality of life and psychiatric symptoms. Ishii et al. (2021) conducted an RCT comparing semaglutide (3, 7, or 14 mg/day) with dulaglutide 0.75 mg weekly in 458 patients over 52 weeks, assessing health‐related quality of life (HRQoL) including anxiety subscale. They found that semaglutide, particularly at 7 and 14 mg, showed more significant improvements in the diabetes therapy‐related quality‐of‐life (DTR‐QoL) scores than dulaglutide. The semaglutide group also had less decline in DTR‐QoL scores from Weeks 26 to 52, indicating sustained benefits. Da Porto et al. (2020) conducted a 12‐week pilot trial with dulaglutide (1.5 mg weekly) in 60 patients with T2DM and BED. They found a significant reduction in binge eating score (BES: 12.067 vs. 0.467, *p *< 0.0001), with improvements linked to changes in body weight (*p *< 0.0001) and HbA1c (*p* = 0.033). However, studies by and Bode et al. (2010) found no significant improvements in depression or anxiety in patients with T2DM receiving liraglutide, highlighting the inconsistency of psychiatric outcomes across studies.

De Wit et al. (2016) and Bode et al. (2010) investigated the effects of liraglutide on depression and anxiety in patients with T2DM but reported no significant improvements. De Wit et al. (2016), in an open‐label trial with 18 patients, administered liraglutide 1.8 mg/day for 26 weeks and found no significant changes in BDI scores (−1 ± 2 vs. 0 ± 1, *p* = 0.46). Similarly, Bode et al. (2010) conducted an RCT in 732 patients, comparing liraglutide doses of 1.2 and 1.8 mg daily over 52 weeks and found no significant impact on HRQoL measures related to depression and anxiety. Ishii et al. (2017) studied liraglutide in an observational study of 204 patients with T2DM over 12 weeks. They found a significant improvement in DTR‐QoL scores from baseline (61.9 ± 16.2) to Week 12 (69.7 ± 16.8, *p *< 0.001), reflecting reductions in anxiety, treatment satisfaction, and daily activity burden.

Li et al. (2023) and reported on the psychiatric side effects of semaglutide in patients with T2DM. Li et al. (2023) reported two cases of depression associated with semaglutide 0.5 mg weekly, the first developed moderate depression, which improved after a week of stopping the medication, whereas the second developed severe depression after 1 month of use, also improving within a week of discontinuation. Similarly, Manoharan et al. (2024) and Rajaram and Madan (2024) observed increased irritability and anxiety in a patient on semaglutide 0.5 mg, with symptoms gradually improving over several weeks after the drug was stopped. In contrast, He et al. (2024) reported a case in which depressive symptoms significantly improved within a week after a patient with T2DM discontinued liraglutide 1.8 mg daily and started antidepressant therapy, with an Hamilton Depression Rating Scale (HAMD) score of 6 and a Hamilton Anxiety Scale score of 5 at discharge; the patient's mood remained stable at a 2‐week follow‐up.

Exenatide has also been studied for its psychiatric effects. Idris et al. (2013) conducted a non‐randomized clinical trial with 16 patients, administering exenatide 5–10 µg twice‐daily for 22 weeks, and observed a significant reduction in BDI scores from baseline to exenatide groups (*p* = 0.001), though no significant difference was observed between placebo and exenatide groups. Grant et al. (2011) in a cohort study with 138 patients found significant improvements in treatment satisfaction (*p *< 0.05) and well‐being (*p *< 0.05) after 6 months of exenatide treatment compared to insulin, with Hospital Anxiety and Depression Scale (HADS) scores that were also significantly reduced in the exenatide group (*p *< 0.05). HADS scores in the GLP‐1‐treated group had a relatively small but significant effect opposed to the insulin group (Grant et al. 2011).

Eren‐Yazicioglu et al. (2021) assessed exenatide in a cross‐sectional study with 43 patients on exenatide 10 µg twice‐daily for at least 3 months. They found that these patients had higher Patient Health Questionnaire‐9 (PHQ‐9) (9.70 ± 4.92 vs. 6.70 ± 4.66; *p* = 0.026) and Perceived Stress Scale (PSS) (29.39 ± 6.70 vs. 23.35 ± 7.69; *p* = 0.015) scores compared to those not on the drug, indicating greater depressive and stress symptoms. Higher PSS scores were also associated with higher PHQ‐9 levels (*b* = 0.236). Best et al. (2011) conducted a double‐dummy trial with 491 patients on exenatide 2 mg weekly for 26 weeks and found improvements in psychological general well‐being (PGWB) scores at Week 26, although there were no statistically significant differences between exenatide and other groups. These findings suggest that the psychiatric effects of exenatide may depend on individual patient factors.

Overall, the psychiatric effects of GLP‐1 receptor agonists in T2DM patients remain inconsistent. Although some cases suggest an association with depressive symptoms and anxiety, others report improved mood and quality of life. The variability in findings highlights the need for further investigation into individual patient factors influencing these effects.

#### 3.3.3 Obesity

Allison et al. (2023) conducted an RCT with 27 patients to examine liraglutide's effects (3.0 mg daily for 12 weeks) on binge eating, depression, and overall quality of life in obesity with BED. By Week 17, liraglutide‐treated participants had reduced objective binge episodes (OBEs) by 4.0 per week, compared to a decrease of 2.5 in the placebo group, though the difference was not statistically significant (*p* = 0.37, mean difference = 1.2). BED remission rates were 44% in the liraglutide group versus 36% in the placebo group, with no significant differences observed in eating behavior, depressive symptoms, or quality of life between the groups. In another RCT, Chao et al. (2019) assessed liraglutide (3.0 mg for 12 weeks) in 150 patients, finding significant improvements in dietary disinhibition, eating disorder psychopathology, and shape concerns among those receiving liraglutide in IBT‐liraglutide and multicomponent groups, compared to the IBT‐alone group. The multicomponent group, in particular, reported greater reductions in binge eating episodes. Lastly, in a clinical trial by Apperley et al. (2021), seven participants on liraglutide (3.0 mg daily for 3 months) demonstrated improvement in anxiety and depressive symptoms (95% CI from −1.11 to 22.11; *p* = 0.068), particularly in features of separation anxiety disorder (95% CI from 0.16 to 20.84; *p* = 0.048).

Richards et al. (2023) conducted a retrospective cohort study with 48 participants to assess the effects of semaglutide on BES in obesity with BED. Results indicated that patients receiving semaglutide alone had greater reductions in BES scores compared to those on combined pharmacotherapy with other anti‐obesity medications, suggesting that semaglutide monotherapy may be more effective for binge eating reduction. Additionally, a case series by Richards et al. (2023), involving six patients on semaglutide (0.25 or 0.5 mg weekly), demonstrated a 100% reduction in AUD symptoms with a mean AUDIT score decrease of 9.5 points (*p *< 0.001), indicating notable improvement in alcohol use symptoms. Kahal et al. (2019) conducted a cross‐sectional study with 36 women with obesity and polycystic ovary syndrome (PCOS) on liraglutide (1.8 mg weekly for 6 months). The findings indicated no significant change in depression scores over the study period, with the proportion of women meeting the Center for Epidemiologic Studies Depression Scale (CES‐D) threshold for depression symptoms decreasing slightly from 32% to 26% in the PCOS group (*p* = 0.72) and from 29% to 18% in the control group (*p* = 0.42).

Overall, semaglutide appears to have a stronger effect on reducing binge eating behaviors compared to liraglutide, with potential benefits for AUD. However, liraglutide's impact on depression and anxiety remains unclear, with studies showing inconsistent outcomes.

#### 3.3.4 Schizophrenia

Ishøy et al. (2017) conducted an RCT with 45 patients with schizophrenia and obesity who received exenatide (2 mg weekly for 3 months). Neurocognition and schizophrenia symptoms improved significantly over time (*p *< 0.001 for Brief Assessment of Cognition in Schizophrenia [BACS] composite score), though there was no significant group effect (*p* = 0.64) or time‐by‐group interaction (*p* = 0.77). Hejdak et al. (2024) described a patient with schizophrenia and T2DM who developed paranoid delusions 2–3 weeks after starting semaglutide (1–2 mg weekly). Delusions resolved after increasing the Geodon dosage but returned upon increasing the semaglutide dose 7 months later.

Although limited data suggest that GLP‐1 receptor agonists may offer cognitive benefits in schizophrenia, semaglutide has been reported to exacerbate psychotic symptoms in one individual. More research is necessary to assess the safety and efficacy of GLP‐1 receptor agonists in this population.

### 3.4 Characteristics of Registered Clinical Trials

#### 3.4.1 Trials by Primary Diagnosis

Out of the clinical trials included in this article, primary diagnoses studied included AUD/alcohol dependence (*n* = 3; 30%), nicotine dependence/addiction (*n* = 2; 20%), a combination of AUD and nicotine dependence (*n* = 2; 20%), BED (*n* = 1; 10%), Opioid‐Related Disorders (*n* = 1; 10%), and Schizophrenia (*n* = 1; 10%) (Table 2).

#### 3.4.2 Trials by Country of Origin and Funding Source

The 10 clinical trial studies included in this article also ranged in country of origin, primarily taking place in the United States (*n* = 7; 70.0%). The additional three clinical trials were conducted in Canada (*n* = 1; 10%), Denmark (*n* = 1; 10%), and the United Kingdom (*n* = 1; 10%). Eight of these clinical trials were funded institutionally, with the remaining two being privately funded.

#### 3.4.3 Trials by Participant Demographics

A total of 632 participants were involved in the 10 clinical trial studies. Out of the 632 participants, 43 completed treatment and the entire trial. The mean age across participants in the clinical trials was 44.03 (SD 9.67). Of the studies that reported participants sex (*n* = 3; 30%), 67% of the participants were female. Three of the 10 included clinical trials implemented a healthy control group in the studies.

#### 3.4.4 Trials by Study Design

Of the 10 included clinical trials, all employed a randomized allocation. Seven (70.0%) utilized parallel group assignment, two (20.0%) utilized crossover group assignment, and one (10.0%) utilized factorial assignment. Regarding masking techniques used in the included trials, six (60.0%) were quadruple‐blinded, in which the patient, care provider, investigator, and outcomes assessor were blinded in the study, and three (30.0%) were triple‐blinded, in which the care provider remained unblinded. The remaining trial was single‐blinded, with only the patient blinded to the intervention.

#### 3.4.5 Trials by Treatment Parameters

Among the included clinical trials, semaglutide was the most commonly administered GLP‐1 receptor agonist (*n* = 5; 50.0%), followed by liraglutide (*n* = 3; 30.0%) and exenatide (*n* = 2; 20.0%). Nine of the 10 included studies employed two study arms investigating the GLP‐1 receptor agonist compared to a placebo injection. The remaining trial used a crossover study design to investigate three study arms: overweight/obese subjects, ex‐smokers and ex‐alcohol‐dependent subjects. The mean study length among the trials was 34 weeks.

#### 3.4.6 Trials by Treatment Efficacy

Three included clinical trials provided study results. One trial investigating symptoms of BED reported the mean value of binge eating episodes per week decreased by 3.97 ± (0.56) among the group treated with liraglutide, compared to a 2.50 ± (0.053) decrease in the placebo group. In one trial investigating alcohol craving and self‐administration, standard drink units consumed decreased to 0.93 ± (0.87) in the group treated with exenatide compared to 2.78 ± (2.66) in the placebo group. Finally, one trial investigating nicotine usage and smoking abstinence reported 10.5% of participants with a 7‐day point prevalence smoking abstinence among the group treated with liraglutide compared to 9.5% in the placebo group at 12 weeks post‐target quit date. This percentage remained the same at 26 weeks post‐target quit date.

## 4 Discussion

This systematic review synthesizes evidence on the psychiatric effects of GLP‐1 RAs, integrating findings across SUDs, metabolic disorders, and comorbid psychiatric conditions. Although studies in SUDs, including CUD, AUD, and nicotine dependence, suggest potential reductions in cravings, inconsistencies in substance use and abstinence outcomes highlight the need to examine underlying neurobiological mechanisms, such as GLP‐1's role in dopamine regulation. In metabolic disorders like T2DM and obesity, improvements in depression, anxiety, and quality of life have been observed, though variability across depression scales suggests heterogeneity in psychiatric effects, potentially influenced by metabolic‐inflammatory pathways. In individuals with both psychiatric and metabolic conditions, such as T2DM, schizophrenia, and BED, findings suggest both beneficial and AEs, including mood instability and psychotic symptoms, emphasizing the need for precision medicine approaches. By integrating these findings, this review highlights critical gaps in understanding GLP‐1 RAs’ psychiatric effects, particularly regarding patient‐specific factors, neurobiological interactions, and potential for clinical translation.

The findings of this review suggest potential differences in the effectiveness of GLP‐1RAs on psychiatric symptoms, depending on whether individuals have primary psychiatric or metabolic conditions. In patients with primary psychiatric disorders, particularly SUDs, the effects of GLP‐1 RAs on cravings and mood were modest and inconsistent, likely reflecting the complex neurobiological underpinnings of psychiatric conditions. Individuals with primary metabolic disorders, such as T2DM or obesity, showed more consistent improvements in depressive symptoms and quality of life with GLP‐1 RAs treatment. This may be because GLP‐1 RAs primarily target metabolic pathways, improving glycemic control and promoting weight loss, which can have secondary positive effects on mood, particularly in conditions where metabolic dysfunctions are linked to psychiatric symptoms (Drew et al. 2022). Improved physical health and energy levels, alongside decreased inflammation and improved insulin sensitivity, could contribute to better mental health outcomes in these patients (Clément et al. 2004). For individuals with comorbid psychiatric and metabolic disorders, the effects of GLP‐1 RAs were more mixed, sometimes leading to mood improvements and other times resulting in adverse psychiatric symptoms, such as mood fluctuations or, in rare cases, psychotic symptoms. This variation might be due to complex bidirectional interactions between metabolic and psychiatric pathways, including inflammatory responses, hypothalamic‐pituitary‐adrenal (HPA) axis dysregulation, and neurotransmitter changes that are difficult to isolate (Tsigos and Chrousos 2002). Given these complexities, future research should focus on characterizing patient subgroups more precisely to identify who may benefit most from GLP‐1 RAs.

Several studies have documented psychiatric AEs associated with GLP‐1 RAs, particularly noting an association between semaglutide and liraglutide with suicidal ideation and self‐injury (McIntyre et al. 2013). Our review also identified cases where semaglutide use coincided with the onset of MDD in two patients—one of whom had a prior history of depression, making it difficult to fully attribute causality to the medication. However, an analysis by McIntyre et al. (2013), using data from the FDA adverse event reporting system (FAERS), found no disproportionate reporting of suicidal behaviors, suicide attempts, or completed suicides for any FDA‐approved GLP‐1 RAs. This underscores the need for rigorous, prospective studies to assess psychiatric risks while accounting for confounding factors such as pre‐existing mood disorders and concurrent medications. Although preliminary evidence suggests potential antidepressant and cognitive benefits, further investigation is needed to determine whether these medications independently influence psychiatric symptoms or if their effects are mediated by improvements in metabolic health. Large‐scale registry studies and biomarker research on inflammation and neuroplasticity could help clarify these mechanisms and guide safe prescribing practices.

The results of the included studies demonstrated a positive safety profile with minimal AEs. Commonly reported AEs included early satiety, abdominal distension, nausea, and fatigue, those of which did not require withdrawal from the studies (Tempia Valenta et al. 2023). Less widely reported AEs included hypertension, acid reflux, abdominal pain, belching, and difficulty concentrating (Isacson et al. 2011). Additionally, mild reactions at injection sites were reported among some patients and one study reported serious complications requiring hospitalizations (Allison et al. 2023). Majority of the studies, however, did not report serious AEs associated with GLP‐1 receptor agonist treatment. Despite the reported absence of serious AEs associated with GLP‐1 receptor agonist administration, the average study duration of the included studies remains 26.4 weeks. Thus, there remains limited knowledge in terms of the long‐term psychiatric effects of the examined GLP‐1 RAs. Importantly, previous animal studies investigating the safety of GLP‐1 RAs for weight loss have reported associations with serious AEs, including pancreatitis, gastroparesis, as well as kidney injury and thyroid cancer in mice (Filippatos et al. 2014); however, this effect has not been replicated in human studies and evidence shows that GLP‐1 RAs provide protective effects, including a reduction in major adverse cardiovascular events, all‐cause mortality, heart failure, and kidney disease (Kristensen et al. 2019). Further study into the long‐term effects of the use of these agonists in psychiatric disorders is required to ensure safety among patients, especially as these therapeutics grow in popularity and in the treatment of psychiatric disorders.

The effect of GLP‐1 RAs in treating psychiatric disorders has been well studied in preclinical models. One study found liraglutide to be effective in reducing corticosterone (CORT)‐induced depressive and anxiety‐like symptoms in mice (Weina et al. 2018). Another study found liraglutide to partially reverse depression‐like behavior as well as metabolic abnormalities when combined with long‐term antipsychotic treatment of olanzapine, which is known to increase body weight as a side effect (Clemente‐Suárez et al. 2023). Scientific evidence exists, through studies implementing animal models, to support the potential benefits of GLP‐1 RAs as a means to combat addiction and substance abuse systems. A study investigating the impact of liraglutide and semaglutide on alcohol intake found significant reductions in voluntary ethanol intake in mice (Sharma et al. 2015); however, it is important to note these results were not maintained longer than 2 days after post‐injection of the receptor agonist drug. Additionally, studies have found GLP‐1 RAs to reduce the effect of cocaine‐mediated behaviors (Sharma et al. 2015). These findings suggest that GLP‐1 RAs may hold promise as adjunctive treatments for psychiatric conditions, but their clinical relevance remains uncertain. Future studies should examine whether metabolic improvements mediate psychiatric benefits or whether GLP‐1 RAs exert direct effects on the brain. Integrating biomarker analyses, neuroimaging, and long‐term psychiatric assessments will be critical to advancing our understanding of their role in neuropsychiatric disorders.

Several hypotheses have been proposed to explain the mechanisms through which GLP‐1 RAs may influence psychiatric disorder symptoms, with a prominent explanation focusing on their role in modulating inflammation and neuroinflammation (Pantovic‐Stefanovic et al. 2024). Evidence underscores a correlation between mood disorders and obesity alongside metabolic factors (Pantovic‐Stefanovic et al. 2024; Kirichenko et al. 2022). In particular, it has been shown that excess adiposity increases systemic inflammation, including release of inflammatory cytokines (e.g., IL‐6, TNF‐alpha) that can infiltrate into the brain, leading to microglial activation and precipitate mood disorders (Kirichenko et al. 2022). This suggests that therapies based on GLP‐1 receptor activation could potentially be relevant in treating mood disorders in a subset of individuals with comorbid obesity or overweight through reducing adiposity and inflammation, as well as promoting neuroplasticity (Kirichenko et al. 2022). Additionally, neuroinflammation is increasingly recognized as a contributing factor to various psychiatric conditions, and targeting this pathway may have therapeutic benefits (Lin et al. 2023). Lin et al. provided compelling evidence for this hypothesis, showing that exenatide, a GLP‐1 receptor agonist, significantly decreased neuroinflammation in mice, which correlated with reduced anxiety‐like behaviors, evidenced by an increased willingness to engage in exploratory activities such as an open field test (Lin et al. 2023). Beyond neuroinflammation, GLP‐1 RAs also protect against neuronal apoptosis, preserving neuronal integrity and function, and they enhance synaptic plasticity by facilitating hippocampal long‐term potentiation (LTP), all of which are critical for cognitive function and may underlie their beneficial effects in psychiatric disorders (Gault 2018).

To our knowledge, the present study is the most comprehensive review that aims to systematically investigate the effect of GLP‐1 RAs on psychiatric symptoms across different populations. First, the majority of studies included varied in sample size, study design, and population characteristics, which may contribute to heterogeneity in the results and limit the generalizability of our conclusions. Additionally, many of the studies relied on self‐reported psychiatric outcomes or AEs, which are subject to recall bias and may not capture the full scope of psychiatric effects associated with GLP‐1 RAs use. The observational nature of most studies also limits our ability to determine causation between GLP‐1 RAs and psychiatric AEs. Moreover, potential confounding factors, such as pre‐existing psychiatric conditions or concurrent use of other medications, were not consistently controlled across studies. These limitations highlight the need for more rigorous, large‐scale RCTs to better assess the psychiatric safety profile of GLP‐1 RAs and to clarify the mechanisms underlying their psychiatric effects. Finally, due to significant heterogeneity among component studies in terms of study design and population, quantitative synthesis was not possible. Moderate‐high quality of included studies is a strength of our review.

## 5 Conclusion

This review highlights the emerging psychiatric effects of GLP‐1 RAs, emphasizing their potential relevance for mood disorders, cognitive dysfunction, and addiction‐related symptoms in individuals with comorbid metabolic conditions. However, findings across studies were mixed, and current evidence remains insufficient to determine whether GLP‐1 RAs have a definitive therapeutic effect on psychiatric symptoms. Some trials demonstrated improvements in mood, reductions in cravings, and potential benefits for individuals with SUDs, whereas others yielded more modest findings in populations with obesity and T2DM. Notably, the potential of GLP‐1 RAs extends beyond weight management, suggesting that their neurobiological effects—particularly on neurotransmitter systems, neuroinflammation, and synaptic plasticity—may contribute to mood stabilization and cognitive enhancement. The novelty of GLP‐1 RAs in the psychiatric realm lies in their multifaceted mechanisms of action, which go beyond traditional pharmacotherapies targeting metabolic pathways. By addressing both neuropsychiatric and metabolic dysfunctions, GLP‐1 RAs represent a promising integrative therapeutic target, yet much more research is needed. Importantly, the present review did not assess publication bias (e.g., through funnel plots), and this may influence the interpretation of available findings. To capitalize on the potential therapeutic value of GLP‐1 RAs, future studies should adopt a more focused and integrative approach, refining the mechanisms through which these agents influence psychiatric symptoms. Although GLP‐1 RAs such as liraglutide, exenatide, and semaglutide showed potential for improving mental health outcomes—particularly depressive symptoms, anhedonia, anxiety, and quality of life—these preliminary signals require confirmation through larger, methodologically rigorous trials. These trials should explore optimal dosing regimens, treatment duration, and target populations, with a particular emphasis on elucidating the neurobiological mechanisms involved. Long‐term impacts on psychiatric conditions also remain unclear and warrant further investigation, particularly in diverse populations with comorbid metabolic disorders.

## Author Contributions

**Shakila Meshkat**: conceptualization, investigation, writing – original draft, writing – review and editing, methodology, formal analysis, project administration. **Corinna Di Luciano**: conceptualization, investigation, writing – original draft, writing – review and editing, methodology, formal analysis, project administration. **Alyssa Swiderski**: conceptualization, investigation, writing – review and editing, writing – original draft, project administration, methodology. **Gloria Li**: writing – review and editing, writing – original draft, conceptualization, methodology. **Reinhard Janssen Aguilar**: writing – original draft, writing – review and editing, conceptualization. **Benjamin T. Dunkley**: writing – review and editing, conceptualization, methodology. **Amy C. Reichelt**: writing – review and editing, conceptualization, methodology. **Yanbo Zhang**: writing – review and editing, conceptualization. **Andrew Greenshaw**: conceptualization, methodology, writing – review and editing. **Eric Vermetten**: methodology, conceptualization, writing – review and editing. **Rakesh Jetly**: methodology, conceptualization, writing – review and editing. **Satya Dash**: methodology, conceptualization, writing – review and editing. **Sri Mahavir Agarwal**: methodology, writing – review and editing, conceptualization. **Jennifer Swainson**: methodology, writing – review and editing, conceptualization. **Venkat Bhat**: methodology, conceptualization, writing – review and editing, writing – original draft, investigation, validation, formal analysis, project administration, supervision, visualization.

## Ethics Statement

The authors have nothing to report.

## Consent

The authors have nothing to report.

## Conflicts of Interest

Jennifer Swainson has received honoraria for speaking or advisory roles from Abbvie, Bausch Health, Biron, Eisai, Idorsia, Janssen, Lundbeck, Novo Nordisk, and Otsuka. Rakesh Jetly is the CMO of Mydecine Innovation Group. Benjamin Dunkley is CSO at MYndspan Ltd, and has received funding from the Department of National Defence (Government of Canada), Canadian Institutes of Health Research, National Institutes of Health and MITACS. SMA has received honoraria from HLS therapeutics and Boehringer‐Ingelheim, Canada, and is supported in part by an Academic Scholar Award from the University of Toronto Department of Psychiatry. Venkat Bhat is supported by an Academic Scholar Award from the University of Toronto Department of Psychiatry and has received research support from the Canadian Institutes of Health Research, Brain & Behavior Foundation, Ontario Ministry of Health Innovation Funds, Royal College of Physicians and Surgeons of Canada, Department of National Defence (Government of Canada), New Frontiers in Research Fund, Associated Medical Services Inc. Healthcare, American Foundation for Suicide Prevention, Roche Canada, Novartis, and Eisai.

## Peer Review

The peer review history for this article is available at https://publons.com/publon/10.1002/brb3.70661

## Supporting information




**Table S1**. Search strategy.
**Table S2**. JBI Quality Assessment.
PRISMA checklist

## Data Availability

The excel sheet for the extracted variables is available upon request.

## References

[brb370661-bib-0001] Acar, A. S. , and O. Erbas . 2021. “2 Glucagon‐Like Peptide‐1 and Psychiatric Disorder.” Journal of Experimental and Basic Medical Sciences 2, no. 2: 106–115. 10.5606/jebms.2021.75645.

[brb370661-bib-0002] Allison, K. C. , A. M. Chao , M. B. Bruzas , et al. 2023. “A Pilot Randomized Controlled Trial of Liraglutide 3.0 mg for Binge Eating Disorder.” Obesity Science & Practice 9, no. 2: 127–136. 37034559 10.1002/osp4.619 PMC10073825

[brb370661-bib-0003] Angarita, G. A. , D. Matuskey , B. Pittman , et al. 2021. “Testing the Effects of the GLP‐1 Receptor Agonist Exenatide on Cocaine Self‐Administration and Subjective Responses in Humans With Cocaine Use Disorder.” Drug and Alcohol Dependence 221: 108614. 33621809 10.1016/j.drugalcdep.2021.108614 PMC8026565

[brb370661-bib-0004] Apperley, L. J. , L. Gait , K. Erlandson‐Parry , P. Laing , and S. Senniappan . 2021. “Liraglutide Combined With Intense Lifestyle Modification in the Management of Obesity in Adolescents.” Journal of Pediatric Endocrinology and Metabolism 34, no. 5: 613–618. 33823101 10.1515/jpem-2020-0714

[brb370661-bib-0005] Best, J. H. , R. R. Rubin , M. Peyrot , et al. 2011. “Weight‐Related Quality of Life, Health Utility, Psychological Well‐Being, and Satisfaction With Exenatide Once Weekly Compared With Sitagliptin or Pioglitazone After 26 Weeks of Treatment.” Diabetes Care 34, no. 2: 314–319. 21270189 10.2337/dc10-1119 PMC3024340

[brb370661-bib-0006] Bode, B. W. , M. A. Testa , M. Magwire , et al. 2010. “Patient‐Reported Outcomes Following Treatment With the Human GLP‐1 Analogue Liraglutide or Glimepiride in Monotherapy: Results From a Randomized Controlled Trial in Patients With Type 2 Diabetes.” Diabetes, Obesity and Metabolism 2, no. 7: 604–612. 10.1111/j.1463-1326.2010.01196.x PMC2901519 20590735

[brb370661-bib-0007] Cabou, C. , and R. Burcelin . 2011. “GLP‐1, the Gut‐Brain, and Brain‐Periphery Axes.” Review of Diabetic Studies: RDS 8, no. 3: 418–431. 22262078 10.1900/RDS.2011.8.418 PMC3280675

[brb370661-bib-0008] Chao, A. M. , T. A. Wadden , O. A. Walsh , et al. 2019. “Effects of Liraglutide and Behavioral Weight Loss on Food Cravings, Eating Behaviors, and Eating Disorder Psychopathology.” Obesity (Silver Spring) 27, no. 12: 2005–2010. 31746553 10.1002/oby.22653 PMC6873814

[brb370661-bib-0009] Clément, K. , N. Viguerie , C. Poitou , et al. 2004. “Weight Loss Regulates Inflammation‐Related Genes in White Adipose Tissue of Obese Subjects.” FASEB Journal 18, no. 14: 1657–1669. 15522911 10.1096/fj.04-2204com

[brb370661-bib-0010] Clemente‐Suárez, V. J. , L. Redondo‐Flórez , A. I. Beltrán‐Velasco , et al. 2023. “The Role of Adipokines in Health and Disease.” Biomedicines 11, no. 5: 1290. 37238961 10.3390/biomedicines11051290 PMC10216288

[brb370661-bib-0011] Da Porto, A. , V. Casarsa , G. Colussi , C. Catena , A. Cavarape , and L. Sechi . 2020. “Dulaglutide Reduces Binge Episodes in Type 2 Diabetic Patients With Binge Eating Disorder: A Pilot Study.” Diabetes & Metabolic Syndrome: Clinical Research & Reviews 14, no. 4: 289–292. 10.1016/j.dsx.2020.03.009 32289741

[brb370661-bib-0012] De Wit, H. M. , G. M. Vervoort , H. J. Jansen , B. E. De Galan , and C. J. Tack . 2016. “Durable Efficacy of Liraglutide in Patients With Type 2 Diabetes and Pronounced Insulin‐Associated Weight Gain: 52‐Week Results From the Effect of Liraglutide on Insulin‐Associated wE Ight GA iN in Patients With Type 2 Diabetes'(ELEGANT) Randomized Controlled Trial.” Journal of Internal Medicine 279, no. 3: 283–292. 26553486 10.1111/joim.12447

[brb370661-bib-0013] Drew, R. J. , P. J. Morgan , C. E. Collins , et al. 2022. “Behavioral and Cognitive Outcomes of an Online Weight Loss Program for Men With Low Mood: A Randomized Controlled Trial.” Annals of Behavioral Medicine 56, no. 10: 1026–1041. 34964449 10.1093/abm/kaab109

[brb370661-bib-0014] Erbil, D. , C. Y. Eren , C. Demirel , M. U. Küçüker , I. Solaroğlu , and H. Y. Eser . 2019. “GLP‐1's Role in Neuroprotection: A Systematic Review.” Brain Injury 33, no. 6: 734–819. 30938196 10.1080/02699052.2019.1587000

[brb370661-bib-0015] Eren‐Yazicioglu, C. Y. , B. Kara , S. Sancak , et al. 2021. “Effect of Exenatide Use on Cognitive and Affective Functioning in Obese Patients With Type 2 Diabetes Mellitus: Exenatide Use Mediates Depressive Scores Through Increased Perceived Stress Levels.” Journal of Clinical Psychopharmacology 41, no. 4: 428–435. 34016830 10.1097/JCP.0000000000001409

[brb370661-bib-0016] Filippatos, T. D. , T. V. Panagiotopoulou , and M. S. Elisaf . 2014. “Adverse Effects of GLP‐1 Receptor Agonists.” Review of Diabetic Studies RDS 11, no. 3: 202–230. 26177483 10.1900/RDS.2014.11.202 PMC5397288

[brb370661-bib-0017] Gault, V. A. 2018. “RD Lawrence Lecture 2017 Incretins: The Intelligent Hormones in Diabetes.” Diabetic Medicine: A Journal of the British Diabetic Association 35, no. 1: 33–40. 29044772 10.1111/dme.13522

[brb370661-bib-0018] Grant, P. , D. Lipscomb , and J. Quin . 2011. “Psychological and Quality of Life Changes in Patients Using GLP‐1 Analogues.” Journal of Diabetes and Its Complications 25, no. 4: 244–246. 21601480 10.1016/j.jdiacomp.2011.03.002

[brb370661-bib-0019] Gupta, T. , M. Kaur , D. Shekhawat , R. Aggarwal , N. Nanda , and D. Sahni . 2023. “Investigating the Glucagon‐Like Peptide‐1 and Its Receptor in Human Brain: Distribution of Expression, Functional Implications, Age‐Related Changes and Species Specific Characteristics.” Basic and Clinical Neuroscience 14, no. 3: 341–353. 38077175 10.32598/bcn.2021.2554.2 PMC10700809

[brb370661-bib-0020] Gupta, V. 2013. “Glucagon‐Like Peptide‐1 Analogues: An Overview.” Indian Journal of Endocrinology and Metabolism 17, no. 3: 413–421. 23869296 10.4103/2230-8210.111625 PMC3712370

[brb370661-bib-0021] He, Y. , F. Liang , Y. Wang , Y. Wei , and T. Ma . 2024. “Liraglutide‐Associated Depression in a Patient With Type 2 Diabetes: A Case Report and Discussion.” Medicine 103, no. 18: e37928. 38701264 10.1097/MD.0000000000037928 PMC11062733

[brb370661-bib-0022] Hejdak, D. , A. N. Razzak , L. Sun , M. Rahman , and P. Jha . 2024. “Interaction of Semaglutide and Ziprasidone in a Patient With Schizophrenia: A Case Report.” Cureus 16, no. 4: e59319. 38817502 10.7759/cureus.59319 PMC11137324

[brb370661-bib-0023] Idris, I. , H. Abdulla , S. Tilbrook , R. Dean , and N. Ali . 2013. “Exenatide Improves Excessive Daytime Sleepiness and Wakefulness in Obese Patients With Type 2 Diabetes Without Obstructive Sleep Apnoea.” Journal of Sleep Research 22, no. 1: 70–75. 22716195 10.1111/j.1365-2869.2012.01030.x

[brb370661-bib-0024] Isacson, R. , E. Nielsen , K. Dannaeus , et al. 2011. “The Glucagon‐Like Peptide 1 Receptor Agonist Exendin‐4 Improves Reference Memory Performance and Decreases Immobility in the Forced Swim Test.” European Journal of Pharmacology 650, no. 1: 249–255. 20951130 10.1016/j.ejphar.2010.10.008

[brb370661-bib-0025] Ishii, H. , B. B. Hansen , J. Langer , and H. Horio . 2021. “Effect of Orally Administered Semaglutide Versus Dulaglutide on Diabetes‐Related Quality of Life in Japanese Patients With Type 2 Diabetes: The PIONEER 10 Randomized, Active‐Controlled Trial.” Diabetes Therapy 12: 613–623. 33460016 10.1007/s13300-020-00985-w PMC7846658

[brb370661-bib-0026] Ishii, H. , T. Niiya , Y. Ono , N. Inaba , H. Jinnouchi , and H. Watada . 2017. “Improvement of Quality of Life Through Glycemic Control by Liraglutide, a GLP‐1 Analog, in Insulin‐Naive Patients With Type 2 Diabetes Mellitus: The PAGE1 Study.” Diabetology & Metabolic Syndrome 9: 1–0. 10.1186/s13098-016-0202-0 PMC5219656 28074109

[brb370661-bib-0027] Ishøy, P. L. , B. Fagerlund , B. V. Broberg , et al. 2017. “No Cognitive‐Enhancing Effect of GLP‐1 Receptor Agonism in Antipsychotic‐Treated, Obese Patients With Schizophrenia.” Acta Psychiatrica Scandinavica 136, no. 1: 52–62. 28260235 10.1111/acps.12711

[brb370661-bib-0028] Kahal, H. , E. Kilpatrick , A. Rigby , A. Coady , and S. Atkin . 2019. “The Effects of Treatment With Liraglutide on Quality of Life and Depression in Young Obese Women With PCOS and Controls.” Gynecological Endocrinology 35, no. 2: 142–145. 30599799 10.1080/09513590.2018.1505848

[brb370661-bib-0029] Kim, Y. K. , O. Y. Kim , and J. Song . 2020. “Alleviation of Depression by Glucagon‐Like Peptide 1 Through the Regulation of Neuroinflammation, Neurotransmitters, Neurogenesis, and Synaptic Function.” Frontiers in Pharmacology 11, no. 101548923: 1270. 32922295 10.3389/fphar.2020.01270 PMC7456867

[brb370661-bib-0030] Kirichenko, T. V. , Y. V. Markina , A. I. Bogatyreva , T. V. Tolstik , Y. R. Varaeva , and A. V. Starodubova . 2022. “The Role of Adipokines in Inflammatory Mechanisms of Obesity.” International Journal of Molecular Sciences 23, no. 23: 14982. 36499312 10.3390/ijms232314982 PMC9740598

[brb370661-bib-0031] Klausen, M. K. , M. E. Jensen , M. Moller , et al. 2022. “Exenatide Once Weekly for Alcohol Use Disorder Investigated in a Randomized, Placebo‐Controlled Clinical Trial.” JCI Insight 7, no. 19: e159863. 36066977 10.1172/jci.insight.159863 PMC9675448

[brb370661-bib-0032] Kristensen, S. L. , R. Rørth , P. S. Jhund , et al. 2019. “Cardiovascular, Mortality, and Kidney Outcomes With GLP‐1 Receptor Agonists in Patients With Type 2 Diabetes: A Systematic Review and Meta‐Analysis of Cardiovascular Outcome Trials.” Lancet Diabetes & Endocrinology 7, no. 10: 776–785. 31422062 10.1016/S2213-8587(19)30249-9

[brb370661-bib-0033] Li, J. R. , J. Cao , J. Wei , and W. Geng . 2023. “Case Report: Semaglutide‐Associated Depression: A Report of Two Cases.” Frontiers in Psychiatry 14, no. 101545006: 1238353. 37706035 10.3389/fpsyt.2023.1238353 PMC10495976

[brb370661-bib-0034] Lin, M. H. , P. C. Cheng , P. J. Hsiao , et al. 2023. “The GLP‐1 Receptor Agonist Exenatide Ameliorates Neuroinflammation, Locomotor Activity, and Anxiety‐Like Behavior in Mice With Diet‐Induced Obesity Through the Modulation of Microglial M2 Polarization and Downregulation of SR‐A4.” International Immunopharmacology 115: 109653. 36587502 10.1016/j.intimp.2022.109653

[brb370661-bib-0035] Lüthi, H. , S. Lengsfeld , T. Burkard , et al. 2024. “Effect of Dulaglutide in Promoting Abstinence During Smoking Cessation: 12‐Month Follow‐Up of a Single‐Centre, Randomised, Double‐Blind, Placebo‐Controlled, Parallel Group Trial.” EClinicalMedicine 68: 102429. 38371479 10.1016/j.eclinm.2024.102429 PMC10873660

[brb370661-bib-0036] Manandhar, B. , and J. M. Ahn . 2015. “Glucagon‐Like Peptide‐1 (GLP‐1) Analogs: Recent Advances, New Possibilities, and Therapeutic Implications.” Journal of Medicinal Chemistry 58, no. 3: 1020–1037. 25349901 10.1021/jm500810s PMC4329993

[brb370661-bib-0037] Mansur, R. B. , J. Ahmed , D. S. Cha , et al. 2017. “Liraglutide Promotes Improvements in Objective Measures of Cognitive Dysfunction in Individuals With Mood Disorders: A Pilot, Open‐Label Study.” Journal of Affective Disorders 207: 114–120. 27721184 10.1016/j.jad.2016.09.056

[brb370661-bib-0038] McIntyre, R. S. , A. M. Powell , O. Kaidanovich‐Beilin , et al. 2013. “The Neuroprotective Effects of GLP‐1: Possible Treatments for Cognitive Deficits in Individuals With Mood Disorders.” Behavioural Brain Research 237, no. 8004872: 164–171. 23000536 10.1016/j.bbr.2012.09.021

[brb370661-bib-0039] Meier, J. J. , and M. A. Nauck . 2005. “Glucagon‐Like Peptide 1 (GLP‐1) in Biology and Pathology.” Diabetes/Metabolism Research and Reviews 21, no. 2: 91–117. 15759282 10.1002/dmrr.538

[brb370661-bib-0040] Mörkl, S. , J. Wagner‐Skacel , T. Lahousen , et al. 2018. “The Role of Nutrition and the Gut‐Brain Axis in Psychiatry: A Review of the Literature.” Neuropsychobiology 79, no. 1: 80–88. 10.1159/000492834 30223263

[brb370661-bib-0041] Müller, T. D. , B. Finan , S. R. Bloom , et al. 2019. “Glucagon‐Like Peptide 1 (GLP‐1).” Molecular Metabolism 30: 72–130. 31767182 10.1016/j.molmet.2019.09.010 PMC6812410

[brb370661-bib-0042] Page, M. J. , J. E. McKenzie , P. M. Bossuyt , et al. 2021. “The PRISMA 2020 Statement: An Updated Guideline for Reporting Systematic Reviews.” BMJ 372: n71. 33782057 10.1136/bmj.n71 PMC8005924

[brb370661-bib-0043] Pantovic‐Stefanovic, M. , M. Velimirovic , V. Jurisic , et al. 2024. “Exploring the Role of TNF‐α, TGF‐β, and IL‐6 Serum Levels in Categorical and Noncategorical Models of Mood and Psychosis.” Scientific Reports 14, no. 1: 23117. 39367011 10.1038/s41598-024-73937-0 PMC11452617

[brb370661-bib-0044] Penn, E. , and D. K. Tracy . 2012. “The Drugs Don't Work? Antidepressants and the Current and Future Pharmacological Management of Depression.” Therapeutic Advances in Psychopharmacology 2, no. 5: 179–188. 10.1177/2045125312445469. 23983973 PMC3736946

[brb370661-bib-0045] Porritt, K. , J. Gomersall , and C. Lockwood . 2014. “JBI's Systematic Reviews: Study Selection and Critical Appraisal.” AJN the American Journal of Nursing 114, no. 6: 47–52. 10.1097/01.NAJ.0000450430.97383.64 24869584

[brb370661-bib-0046] Probst, L. , S. Monnerat , D. R. Vogt , et al. 2023. “Effects of Dulaglutide on Alcohol Consumption During Smoking Cessation.” JCI Insight 8, no. 22: e170419. 37991022 10.1172/jci.insight.170419 PMC10721313

[brb370661-bib-0047] Rajaram, M. S. V. , and R. Madan . 2024. “GLP‐1 Agonists Can Affect Mood: A Case of Worsened Depression on Ozempic (Semaglutide).” Innovations in Clinical Neuroscience 21, no. 4–6: 25–26. PMC11208009 38938530

[brb370661-bib-0048] Richards, J. , N. Bang , E. L. Ratliff , et al. 2023. “Successful Treatment of Binge Eating Disorder With the GLP‐1 Agonist Semaglutide: A Retrospective Cohort Study.” Obesity Pillars Online 7, no. 9918697364706676:100080. 10.1016/j.obpill.2023.100080 PMC10661993 37990682

[brb370661-bib-0049] Richards, J. R. , M. F. Dorand , K. Royal , L. Mnajjed , M. Paszkowiak , and W. K. Simmons . 2023. “Significant Decrease in Alcohol Use Disorder Symptoms Secondary to Semaglutide Therapy for Weight Loss: A Case Series.” Journal of Clinical Psychiatry 85, no. 1: 23m15068. 10.4088/JCP.23m15068 38019594

[brb370661-bib-0050] Robert, S. A. , A. G. Rohana , S. A. Shah , K. Chinna , W. N. Wan Mohamud , and N. A. Kamaruddin . 2015. “Improvement in Binge Eating in Non‐Diabetic Obese Individuals After 3 Months of Treatment With Liraglutide—A Pilot Study.” Obesity Research & Clinical Practice 9, no. 3: 301–304. 25870084 10.1016/j.orcp.2015.03.005

[brb370661-bib-0051] Saarni, S. I. , J. Suvisaari , H. Sintonen , et al. 2007. “Impact of Psychiatric Disorders on Health‐Related Quality of Life: General Population Survey.” British Journal of Psychiatry 190, no. 4: 326–332. 10.1192/bjp.bp.106.025106 17401039

[brb370661-bib-0052] Sharma, A. N. , A. Pise , J. N. Sharma , and P. Shukla . 2015. “Glucagon‐Like Peptide‐1 (GLP‐1) Receptor Agonist Prevents Development of Tolerance to Anti‐Anxiety Effect of Ethanol and Withdrawal‐Induced Anxiety in Rats.” Metabolic Brain Disease 30, no. 3: 719–730. 25380665 10.1007/s11011-014-9627-z

[brb370661-bib-0053] Tempia Valenta, S. , M. Stecchi , and F. Perazza , et al. 2023. “Liraglutide 3.0 mg and Mental Health: Can Psychiatric Symptoms be Associated to Adherence to Therapy? Insights From a Clinical Audit.” Eating and Weight Disorders: EWD 28, no. 1: 99. 38015342 10.1007/s40519-023-01625-5 PMC10684642

[brb370661-bib-0054] Tsigos, C. , and G. P. Chrousos . 2002. “Hypothalamic–Pituitary–adrenal Axis, Neuroendocrine Factors and Stress.” Journal of Psychosomatic Research 53, no. 4: 865–871. 12377295 10.1016/s0022-3999(02)00429-4

[brb370661-bib-0055] Weina, H. , N. Yuhu , H. Christian , L. Birong , S. Feiyu , and W. Le . 2018. “Liraglutide Attenuates the Depressive‐ and Anxiety‐Like Behaviour in the Corticosterone Induced Depression Model via Improving Hippocampal Neural Plasticity.” Brain Research 1694, no. 0045503: 55–62. 29705602 10.1016/j.brainres.2018.04.031

[brb370661-bib-0056] Yammine, L. , J. C. Balderas , M. F. Weaver , and J. M. Schmitz . 2023. “Feasibility of Exenatide, a GLP‐1R Agonist, for Treating Cocaine Use Disorder: A Case Series Study.” Journal of Addiction Medicine 17, no. 4: 481–484. 37579116 10.1097/ADM.0000000000001147

